# Evolution of *UCP1* Transcriptional Regulatory Elements Across the Mammalian Phylogeny

**DOI:** 10.3389/fphys.2017.00670

**Published:** 2017-09-20

**Authors:** Michael J. Gaudry, Kevin L. Campbell

**Affiliations:** Department of Biological Sciences, University of Manitoba Winnipeg, MB, Canada

**Keywords:** uncoupling protein 1, evolution, transcriptional regulation, enhancer, comparative analysis

## Abstract

Uncoupling protein 1 (UCP1) permits non-shivering thermogenesis (NST) when highly expressed in brown adipose tissue (BAT) mitochondria. Exclusive to placental mammals, BAT has commonly been regarded to be advantageous for thermoregulation in hibernators, small-bodied species, and the neonates of larger species. While numerous regulatory control motifs associated with *UCP1* transcription have been proposed for murid rodents, it remains unclear whether these are conserved across the eutherian mammal phylogeny and hence essential for UCP1 expression. To address this shortcoming, we conducted a broad comparative survey of putative *UCP1* transcriptional regulatory elements in 139 mammals (135 eutherians). We find no evidence for presence of a *UCP1* enhancer in monotremes and marsupials, supporting the hypothesis that this control region evolved in a stem eutherian ancestor. We additionally reveal that several putative promoter elements (e.g., CRE-4, CCAAT) identified in murid rodents are not conserved among BAT-expressing eutherians, and together with the putative regulatory region (PRR) and CpG island do not appear to be crucial for UCP1 expression. The specificity and importance of the upTRE, dnTRE, URE1, CRE-2, RARE-2, NBRE, BRE-1, and BRE-2 enhancer elements first described from rats and mice are moreover uncertain as these motifs differ substantially—but generally remain highly conserved—in other BAT-expressing eutherians. Other *UCP1* enhancer motifs (CRE-3, PPRE, and RARE-3) as well as the TATA box are also highly conserved in nearly all eutherian lineages with an intact *UCP1*. While these transcriptional regulatory motifs are generally also maintained in species where this gene is pseudogenized, the loss or degeneration of key basal promoter (e.g., TATA box) and enhancer elements in other *UCP1*-lacking lineages make it unlikely that the enhancer region is pleiotropic (i.e., co-regulates additional genes). Importantly, differential losses of (or mutations within) putative regulatory elements among the eutherian lineages with an intact *UCP1* suggests that the transcriptional control of gene expression is not highly conserved in this mammalian clade.

## Introduction

Uncoupling protein 1 (UCP1) expression is a defining characteristic of brown adipose tissue (BAT), allowing this specialized eutherian heater organ to function in non-shivering thermogenesis (NST). UCP1 spans the mitochondrial inner-membrane of brown adipocytes, acting to promote mitochondrial proton leak, which dissipates the electrochemical gradient that typically drives ATP synthase. In an effort to defend the mitochondrial proton motive force, the electron transport chain thus pumps protons into the inter-membrane space at an elevated rate via an increased level of substrate combustion, thereby resulting in substantial heat production in the form of NST (Cannon and Nedergaard, 2004; Klingenspor and Fromme, 2012).

Vital to its function, BAT is highly vascularized and localized primarily to the thoracic region, lying adjacent to major blood vessels of the heart (e.g., the Sulzer's vein) permitting effective transfer of NST heat to the rest of the body via the circulatory system (Klingenspor and Fromme, 2012; Oelkrug et al., 2015). This provides a more efficient means of heat production than shivering thermogenesis, which has major drawbacks as it impedes locomotion and produces heat in large muscle groups of the limbs that are prone to heat loss due to their high surface area to volume ratios (Oelkrug et al., 2015). For these reasons, UCP1 is widely considered to have provided a key thermoregulatory and evolutionary advantage to the eutherian lineage, particularly for small-bodied and hibernating species, and, while BAT in larger-bodied species (e.g., humans) is typically lost with the onset of adulthood, it has been generally understood to play vital role in their neonates (Cannon and Nedergaard, 2004).

The *UCP1* gene predates the divergence of ray- and lobe-finned fishes (420 million years ago [MYA]) and can be distinguished from *UCP2* and *UCP3* paralogs by its conserved synteny among vertebrates, as *UCP1* is flanked by the upstream *TBC1D9* and downstream *ELMOD2* loci (Jastroch et al., 2008; Klingenspor et al., 2008). UCP2 and UCP3 have been long-believed to play non-thermogenic roles, and are instead hypothesized to perform a multitude of functions including the reduction of reactive oxygen species by promoting a low level of mitochondrial proton leak when activated by fatty acids (Brand and Esteves, 2005; Echtay, 2007; Mailloux and Harper, 2011). However, a recent study by Lin et al. (2017) suggests that proton uncoupling by UCP3 permits heat production in beige adipose tissue of pigs, compensating for the loss of UCP1 in this lineage (Berg et al., 2006). Nevertheless, the functional roles of both UCP2 and UCP3 remain hotly debated. Similarly, the ancestral function of UCP1 in non-eutherians is currently unclear (Klingenspor et al., 2008). UCP1 expression has been shown to increase with cold exposure in common carp (*Cyprinus carpio*) brain tissue, suggesting a possible role in local thermogenesis (Jastroch et al., 2007). However, to date, this protein has not been definitively linked to heat production in ectothermic vertebrates (Jastroch et al., 2007). While the fat-tailed dunnart (*Sminthopsis crassicaudata*), a marsupial, displays a primitive “brownish” interscapular adipose depot that up-regulates UCP1 expression in response to cold exposure (Jastroch et al., 2008), this tissue is incapable of adaptive NST (Polymeropoulos et al., 2012) with no study demonstrating that UCP1 contributes to NST in marsupials. Although *UCP1* appears to have been inactivated early in the evolution of the eutherian superorder Xenarthra (Gaudry et al., 2017), BAT-mediated adaptive thermogenesis is widely known to occur in small-bodied members of the superorders Laurasiatheria and Euarchontoglires (Oelkrug et al., 2015), and has been documented in the rock elephant shrew (*Elephantulus myurus*; Mzilikazi et al., 2007) and the lesser hedgehog tenrec (*Echinops telfairi*; Oelkrug et al., 2013), both members of the eutherian superorder Afrotheria. These observations strongly suggest that UCP1 was recruited for BAT-mediated NST in a common eutherian ancestor by gain of function mutations in the amino acid sequence of the protein and/or greater control over gene transcription that allowed highly concentrated UCP1 expression within BAT mitochondria (Klingenspor et al., 2008).

**Figure 1:**
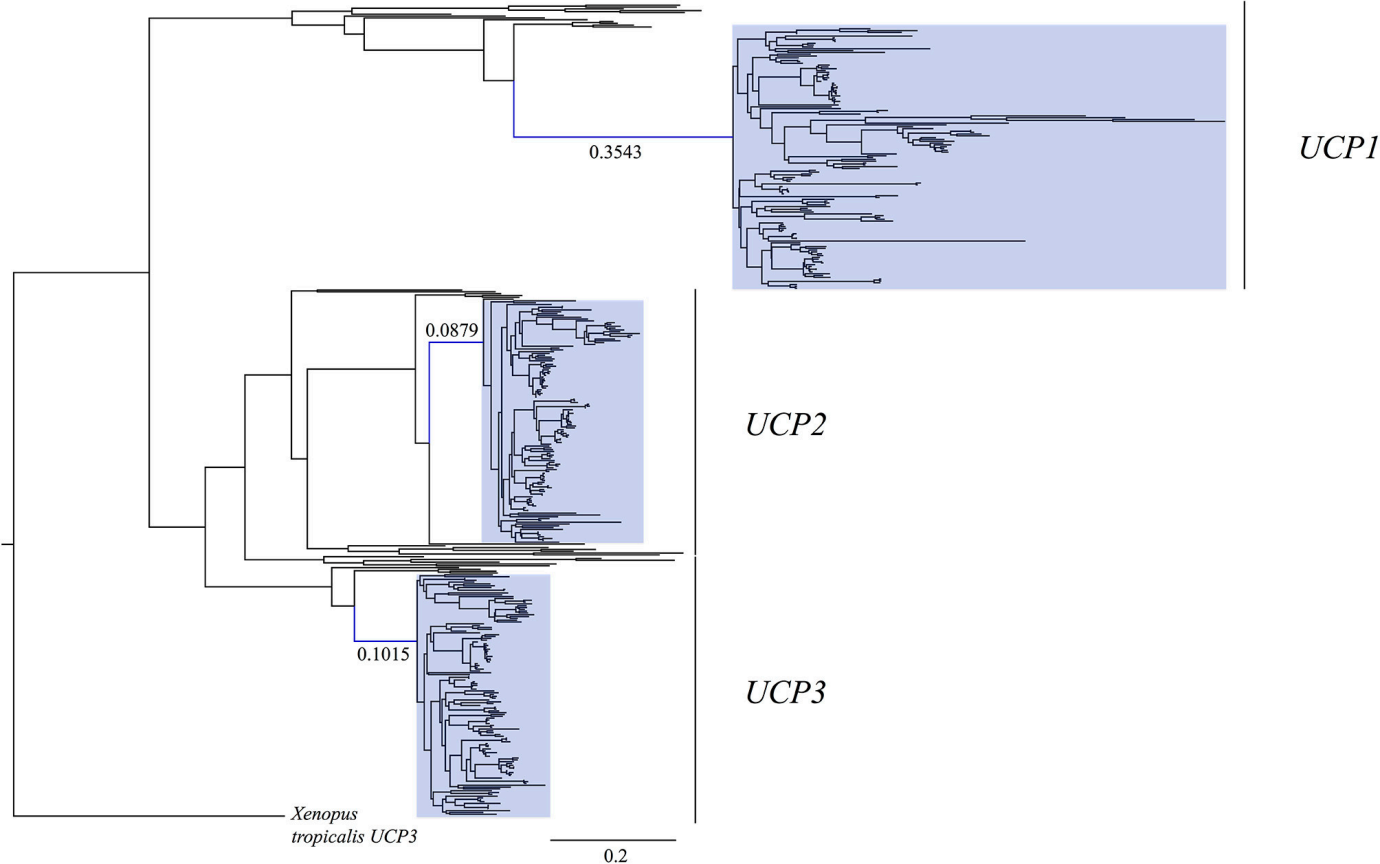
Maximum likelihood gene tree of *UCP1, UCP2*, and *UCP3* coding sequences (*N* = 448) modified from Gaudry et al. (2017) to include the 16 additional species with recently available genome projects (see Table 1). The stem placental mammal branches are indicated in blue. Note that the *UCP1* stem placental branch is much longer than those of *UCP2* and *UCP3*, demonstrating a greater number of nucleotide substitutions per site. Placental mammal genes are highlighted with blue boxes. The tree was rooted with the western clawed frog (*Xenopus tropicalis*) *UCP3*.

Consistent with the gain of function hypothesis, comparative phylogenetic analyses reveal that the stem eutherian branch is highly elongated in *UCP1* gene trees relative to that of *UCP2* and *UCP3* paralogs (Saito et al., 2008; Hughes et al., 2009; Gaudry et al., 2017; Figure 1). It is thus likely that an elevated rate of non-synonymous *UCP1* nucleotide substitutions in the stem eutherian branch conferred this protein with the ability to facilitate proton leak at physiologically significant levels (Jastroch et al., 2008; Klingenspor et al., 2008). While Saito et al. (2008) first proposed *UCP1* evolved under positive selection in basal eutherians, more recent selection pressure analyses reveal non-synonymous to synonymous substitution ratios (dN/dS or ω) of ~0.5–0.6 that are more consistent with relaxed purifying selection (Hughes et al., 2009; Gaudry et al., 2017). However, given that UCP1 of placental mammals possess several unique amino acids relative to non-eutherians, it is possible that directional selection was limited to certain codons along the stem eutherian branch, though, so far this hypothesis remains statistically unsupported (Hughes et al., 2009; Gaudry et al., 2017).

Along with the increased rate of UCP1 evolution in stem eutherians, expression of this protein also became highly tissue-specific during the rise of BAT (Cannon and Nedergaard, 2004). In contrast to the seemingly constitutive presence of UCP1 in common carp brain, liver, and kidney tissues (Jastroch et al., 2007), eutherian UCP1 expression is tightly regulated, occurring predominantly in BAT (Cannon and Nedergaard, 2004). One notable exception, however, is the recently discovered “beige or brite (brown in white)” adipocytes in rodents (mice and rats) and humans. These are derived from white adipose cells that, upon cold exposure, become BAT-like by expressing UCP1 and by having multilocular lipid droplets and an elevated mitochondrial concentration (Harms and Seale, 2013). An important distinction in BAT (and *UCP1*) evolution is that BAT-dependent NST relies upon exceptionally high levels of UCP1 expression, constituting up to 10% of the mitochondrial membrane proteins, whereas UCP2 and UCP3 expression is several orders of magnitude lower (0.01–0.1%) in other tissues (Brand and Esteves, 2005). Interestingly, an enhancer box has been well documented to play a major role in eutherian *UCP1* gene transcription, but is absent in the gray short-tailed opossum (*Monodelphis domestica*; Jastroch et al., 2008), suggesting that it originated with the advent of eutherian UCP1-mediated NST, thus highlighting the importance that gene regulation likely played in the rise of eutherian BAT-mediated thermogenesis.

Given the thermoregulatory advantages conferred by BAT, it is believed that this tissue was fundamental to the evolutionary success of eutherian mammals, and it has even been hypothesized to underlie their colonization of cold ecological niches (Cannon and Nedergaard, 2004). The documented inactivation of the *UCP1* gene in suids (pigs) (Berg et al., 2006) initially emphasized the importance of BAT-mediated thermogenesis, as this inactivation appears to have had detrimental consequences as newborn piglets are widely known to have meager thermoregulatory abilities, suffering from high infant mortality when cold-stressed and relying upon shivering thermogenesis and maternal nest-building in order to maintain homeothermy (Herpin et al., 2002; Berg et al., 2006). By contrast, two recent studies (Gaudry et al., 2017; McGaugh and Schwartz, 2017) contested the conventional belief regarding the importance of BAT-mediated NST throughout the course of placental evolution. Indeed, Gaudry et al. (2017) not only detailed ancient pseudogenization events of *UCP1* in eight additional eutherian lineages: Equidae (horses), Cetacea (whales and dolphins), Proboscidea (elephants and mammoths), Sirenia (sea cows), Hyracoidea (hyraxes), Pholidota (pangolins), Pilosa (sloths and anteaters), and Cingulata (armadillos), but concluded that extreme cold tolerance evolved in many of these groups in the absence of UCP1-mediated thermogenesis.

With the exception of xenarthrans and pangolins, who have adopted a strategy of reduced energy expenditure (i.e., low metabolic rates and body temperatures) associated with their low energy diets, and pigs, for which no credible explanation for *UCP1* inactivation has yet been put forward, Gaudry et al. (2017) proposed that *UCP1* inactivations date back to a period of substantial planetary cooling ~55 to 22 MYA that triggered pronounced increases in body size in other UCP1-lacking lineages (Gaudry et al., 2017). The inverse relationship between the surface-area-to-volume ratio and size imparts greater retention of heat in larger bodied mammals, thus larger mammals have proportionally lower rates of heat production per gram of body mass (McNab, 1983). This linkage is reflected in the diminishing fraction of eutherian body mass constituted by BAT, as well as a reduced NST capacity, with increasing body size (Heldmaier, 1971; Oelkrug et al., 2015). Heldmaier (1971) further suggested that BAT-mediated NST is negligible for mammals >10 kg. Nonetheless, several large-bodied taxa retain an intact *UCP1* gene (e.g., rhinoceroses, pinnipeds, hippopotamus, and camel; Gaudry et al., 2017). Despite this finding, it remains conceivable that members of these groups do not express *UCP1* in BAT, even as neonates. For example, Rowlatt et al. (1971) noted the absence of BAT upon examination of a single newborn hippopotamus (*Hippopotamus amphibious*), while both UCP1 expression and discernable BAT was not detected in either Weddell seal (*Leptonychotes weddellii*) or hooded seal neonates (*Cystophora cristata*) (Pearson et al., 2014). Additionally, the Bactrian camel (*Camelus ferus*) *UCP1* gene displays a 12 base pair nucleotide deletion in exon 5 that would impart the loss of 4 amino acids in close proximity to a site that putatively binds GDP to act as a regulator (inhibitor) of protein activity (Gaudry et al., 2017). Consequently, disruptions to *UCP1* regulatory regions may preclude expression of this protein in BAT of these lineages.

### Evolution of eutherian *UCP1* regulatory elements

**Figure 2:**
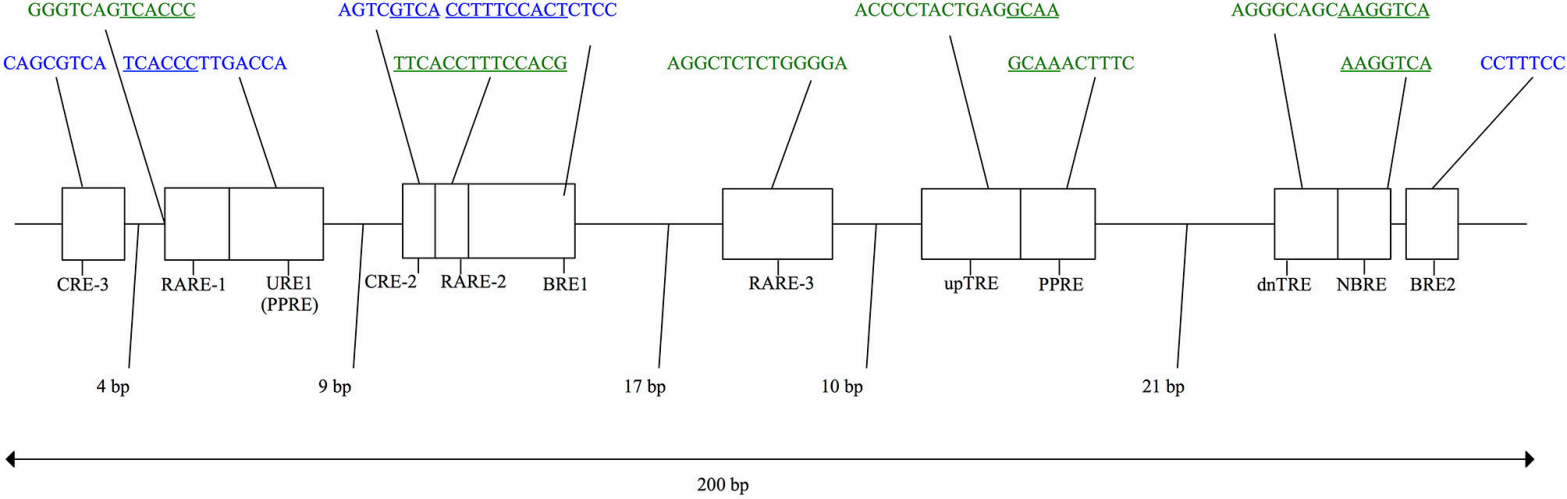
Schematic of the murid *UCP1* enhancer with putative transcription factor binding motifs shown for the rat (green) and mouse (blue) based on a combination of previous studies (see text for details). Regions of overlap between adjacent transcription factor motifs are underlined.

In eutherian mammals, the neuro-hormonal modulation and tissue-specific expression of *UCP1* is under the control of two regulatory regions in the 5′ non-coding region of the gene—a complex distal enhancer region and a proximal promoter—through their interactions with a broad assemblage of transcription factors (Villarroya et al., 2017). Based primarily on murid rodent studies, several putative transcription factor binding motifs (see Figure 2) have been proposed within a conserved ~200 bp *UCP1* enhancer box located ~2–5 kb upstream of the transcriptional start site in eutherians (Cannon and Nedergaard, 2004; Jastroch et al., 2008; Shore et al., 2012). For instance, two cAMP response elements (CREs) were discovered in mice and termed “CRE-3” and “CRE-2” (Kozak et al., 1994). CRE sites typically have a palindromic consensus sequence of 5′-T(G/T)ACGTCA-3′ (Bokar et al., 1988; Kozak et al., 1994). While the first three nucleotides of the two mouse CREs deviate from the typical consensus sequence (Figure 2), the 5′-CGTCA-3′ nucleotides remain conserved and are believed to be key for *UCP1* expression. Indeed, site-directed mutagenesis of these nucleotides within the enhancer CRE of glycoprotein hormone and phosphoenolpyruvate carboxykinase genes has been shown to drastically reduce transcription factor (i.e., cAMP response element binding protein [CREB]) binding and expression in human and rat cells (Bokar et al., 1988). Two “brown adipocyte regulatory element” (BRE) protein-binding motifs (Kozak et al., 1994) also occur in the mouse *UCP1* enhancer box (Figure 2). Again, site directed mutagenesis of the “TTCC” nucleotides within the BREs to a “GTAC” sequence drastically reduces *UCP1* enhancer activity measured using transient expression assays (Kozak et al., 1994). In addition, Sears et al. (1996) found a stretch of nucleotides they termed “UCP regulatory element 1” (URE1), though this is referred to as the peroxisome proliferator response element (PPRE) by Jastroch et al. (2008); Jastroch also predicted a second possible PPRE motif downstream of the URE1 (PPRE) site. The URE1 motif displays high similarity to DR-1 elements (Sears et al., 1996), which are known to comprise of two direct repeats of the “AGGTCA” half-site consensus sequence separated by a single nucleotide (hence the term DR-1; i.e., direct repeats separated by 1 spacer nucleotide). In mice this sequence occurs in the reverse and complement orientation of the first DNA strand (5′- TCACCCTTGACCA-3′), and although it is not an exact match to the consensus sequence, it has been shown to bind the peroxisome proliferator-activated receptor γ and retinoid X receptor α (PPARγ-RXRα) heterodimer transcription factor (Sears et al., 1996). Conversely, mutant variants of the URE1 sequence (i.e., 5′-TCACAATTGACCA-3′ or 5′-TCACCCTAGACCA-3′) failed to bind the PPARγ-RXRα transcription factor, suggesting a key role in the functionality of the *UCP1* enhancer (Sears et al., 1996). Additionally, in light of the requirement of triiodothyronine (T3) for proper BAT expression (Bianco and Silva, 1987), Rabelo et al. (1995) described two putative thyroid hormone response elements (TREs) in the rat *UCP1* enhancer termed “upTRE” and “dnTRE” (Figure 2). TREs typically include two or more variations of the “AGGT(C/A)A” half-site consensus sequence separated by four nucleotides (Brent et al., 1991; Umesono et al., 1991). This same half-site sequence was mentioned above for URE1 and is indeed recognized by multiple transcription factors (Brent et al., 1991). Mutations of the 3′ portion of the upTRE (5′-AGGCAA-3′) and the dnTRE (5′-AGGTCA-3′) to “5′-ATTTAA-3′” and “5′-ATATTA-3′”, respectively, eliminate T3 receptor interactions with the rat *UCP1* enhancer (Rabelo et al., 1995). Three putative retinoic acid response elements (RAREs) within the rat *UCP1* enhancer have also been described by Rabelo et al. (1996), though both RARE-1 and RARE-2 overlap with other binding motifs (see Figure 2). Nonetheless, mutations increasing the AT-richness of these former regulatory elements were shown to significantly disrupt retinoic acid receptor (RAR) and retinoid X receptor (RXR) transcription factor binding (Rabelo et al., 1996). Finally, Kumar et al. (2008) noted a putative nerve growth factor response element (NBRE) within the *UCP1* enhancer of mice (Figure 2) that binds nuclear receptors 4A (NR4A), which acts to promote gene transcription. In addition to the enhancer box, Shore et al. (2012) described a 678 bp putative regulatory region (PRR) located 2,095 bp upstream of the transcriptional start site in humans that was conserved in 14 of 25 of the eutherian species they examined. While Shore et al. (2012) found no evidence that this conserved region plays a role in *UCP1* expression, they did note that it encompassed several possible transcription factor binding motifs, including DR1, DR3, DR4, CEBP (CCAAT-enhancer-binding proteins), CREB, and PPAR.

Transcriptional control of the *UCP1* gene has also been hypothesized to be regulated by a basal promoter occurring within ~250 bp upstream of the transcription start site (Shore et al., 2010). Within this region, Bouillaud et al. (1988) identified a putative TATA box and a CCAAT binding site located ~20 and ~30 bp upstream of the transcriptional start site of the rat *UCP1* gene, respectively. Generally, the TATA box consists of an A/T-rich consensus sequence (5′-TATAAAA-3′; Xu et al., 1991) that interacts with the TATA binding protein (TBP), one of the components of the transcription factor IID (TFIID) that initiates transcription via RNA polymerase II (Nakajima et al., 1988; Patikoglou et al., 1999). The promoters of some mammalian genes (e.g., globins) also contain a CCAAT box typically situated –60 to –100 bp upstream of the transcription start site that binds nuclear transcription factor Y (NF-Y) subunit or CCAAT/enhancer binding protein (C/EBP), which then aids in the initiation of transcription via RNA polymerase II (Mantovani, 1999). Additionally, a putative CRE site (termed CRE-4) occurs ~130 bp upstream of the mouse *UCP1* transcriptional start site in a reverse and compliment orientation (5′-TGACGCGC-3′), with mutations to this sequence eliminating 90–95% of reporter gene expression (Kozak et al., 1994). Yubero et al. (1994) further noted three GCCCCT sequences occurring within ~210 bp of the transcriptional start site of the rat, which DNAse 1 footprinting analyses suggest interact with nuclear proteins found within BAT cells, but these have not been defined as protein binding motifs.

Finally, a CpG island surrounding the *UCP1* proximal promoter and extending into exon 1 has been described in several eutherian species (Kiskinis et al., 2007; Shore et al., 2010, 2012). CpG islands contain high densities of cytosine (C) and guanine (G) nucleotide pairs occurring in the 5′ to 3′ direction and linked by a phosphate (i.e., 5′–C–phosphate–G–3′). These CpG dinucleotides are uncommon in vertebrate genomes, typically occurring at only 20–25% of the frequency anticipated by random chance and act as DNA methylation sites that can modulate gene transcription (Gardiner-Garden and Frommer, 1987). Located immediately upstream of many housekeeping genes, CpG islands are believed to play a major role in their transcriptional control (Gardiner-Garden and Frommer, 1987). Indeed, methylation of CpG dinucleotides immediately upstream of the *UCP1* gene have been shown to modulate gene activity by blocking transcription, whereas demethylation promotes transcription (Shore et al., 2010). Thus, this CpG island has been postulated to be important for *UCP1* gene regulation and, potentially, tissue specific expression within BAT (Kiskinis et al., 2007; Shore et al., 2010).

Because the majority of studies investigating the transcriptional control of *UCP1* have focused on rodents, the status of these transcription factor binding motifs in other eutherian species remain largely unexplored. Here we use genome mining and hybridization-capture techniques coupled with next-generation sequencing to identify and examine *UCP1* transcriptional regulatory elements in 139 mammals (135 eutherians). Briefly, putative transcription factor binding motifs and CpG islands were evaluated using a comparative approach to first determine if they are universally conserved among eutherian superorders with functional BAT, and second to test if they are mutated or lost in large-bodied species that presumably have little or no need for NST. We further anticipated that crucial DNA motifs involved in *UCP1* transcription would have deteriorated via millions of years of neutral evolution in the nine lineages for which *UCP1* has been inactivated.

## Materials and methods

### *UCP1* regulatory sequences

In total, *UCP1* upstream regions of 139 mammals (1 monotreme, 3 marsupials, 3 xenarthrans, 11 afrotherians, 65 laursiatherians, and 56 euarchontoglires) were examined for transcriptional regulatory elements (see Table 1 for species list). This data set employed 116 species whose *UCP1* loci were previously annotated by Gaudry et al. (2017) together with 16 additional species whose genomes have recently been sequenced (denoted by asterisks in Table 1). Regulatory elements of seven additional eutherians were also retrieved by hybridization capture and next-generation sequencing techniques. Briefly, *UCP1* enhancers, PRRs, and basal promoters of four rhinoceroses (black rhinoceros: *Diceros bicornis*, Indian rhinoceros: *Rhinoceros unicornis*, Sumatran rhinoceros; *Dicerorhinus sumatrensis*, and woolly rhinoceros; *Coelodonta antiquitatis*), one tapir (Malayan tapir; *Tapirus indicus*), and two sirenians (dugong; *Dugong dugon*, and Steller's sea cow; *Hydrodamalis gigas*), were targeted using hybridization capture and next-generation sequencing techniques (Springer et al., 2015; Gaudry et al., 2017). Barcoded rhinoceros DNA libraries were constructed using NEBNext Fast DNA Library Prep Set for Ion Torrent and NEBNext DNA Library Prep Master Mix Set for 454 kits (New England Biolabs; Ipswich, Massachusetts, USA) and target-enriched using MyBaits (Mycroarray; Ann Arbor, Michigan, USA) 120mer RNA probes designed to capture *UCP1* exons and regulatory elements based on the orthologous sequences of the white rhinoceros (*Ceratotherium simum*) genome. The captured rhinoceros reads were sequenced on an Ion Torrent PGM platform using Ion 314 v2 and Ion 318 v2 barcoded chips and an Ion PGM Hi-Q sequencing kit (Applied Biosystems; Foster City, California, USA). Sirenian DNA libraries prepared following the methods of Meyer and Kircher (2010) were enriched using an Agilent SureSelect Capture array with probes designed from African elephant (*Loxodonta africana*) *UCP1* upstream sequences. Sirenian DNA reads were sequenced on Illumina GAIIx and HiSeq2500 (Illumina Inc.; San Diego, California, USA) platforms. Sequenced reads were assembled to reference sequences of the white rhinoceros or manatee (*Trichechus manatus*) using the “map to reference” feature in Geneious R9.1 (Biomatters Ltd.; Auckland, New Zealand) at 20% maximum mismatch per read and consensus sequences were generated.

**Table 1:** Presence and absence of the *UCP1* enhancer, putative regulatory region (PPR), and CpG island in 139 mammalian species.

**Species name**	**Enhancer**	**PPR**	**CPG island**	**Accession number**
**MONTREMATA**				
*Ornithorhynchus anatinus*	X	X	X	NW_001794248.1
**MARSUPIALIA**				
*Monodelphis domestica*	X	X	X	AAFR03015618.1
*Macropus eugenii*	/	/	X	ABQO020217652.1
*Sarcophilus harrisii*	/	/	X	AEFK01228715.1
**XENARTHRA**				
*Choloepus hoffmanni*	X	/	/	
*Dasypus novemcinctus*	X	X	Yes	AAGV03181320.1
*Mylodon darwinii*	Yes	/	/	SRX327588
**AFROTHERIA**				
*Chrysochloris asiatica*	Yes	Yes	X	AMDV01244955.1
*Dugong dugon*	Yes	Yes	/	MF871621
*Echinops telfairi*	Yes	Yes	Yes	AAIY02209271.1
*Elephantulus edwardii*	Yes	Yes	X	AMGZ01097263.1
*Elephas maximus*	Yes	Yes	Yes	SRX1015608; SRX1015606; SRX1015604; SRX1015603
*Hydrodamalis gigas*	Yes	/	/	MF871622
*Loxodonta africana*	Yes	Yes	Yes	AAGU03034821.1
*Mammuthus primigenius*	Yes	Yes	/	SRX1015727; SRX1015732; SRX1015743; SRX1015748; SRX001906; ERP008929
*Orycteropus afer*	Yes	Yes	X	ALYB01104541.1
*Procavia capensis*	X	Yes	Yes	ABRQ02143236.1
*Trichechus manatus latirostris*	Yes	Yes	Yes	AHIN01109623.1
**LAURASIATHERIA**				
*Acinonyx jubatus*	Yes	Yes	Yes	LLWD01000416.1
*Ailuropoda melanoleuca*	Yes	Yes	Yes	LNAT01000144.1
*Balaena mysticetus*	Yes	Yes	Yes	SRX790318, SRX790317, SRX790316, SRX790303, SRX790319
*Balaenoptera acutorostrata*	Yes	Yes	Yes	ATDI01065547.1
*Balaenoptera bonaerensis*	Yes	Yes	Yes	BAUQ01197845.1
*Balaenoptera physalus*	Yes	Yes	Yes	SRX1571086, SRX323050
*Bison bison*	Yes	Yes	Yes	JPYT01100523.1
*Bos grunniens*	Yes	Yes	Yes	AGSK01075302.1
*Bos indicus*	/	Yes	/	AGFL01142554.1
*Bos taurus*	Yes	Yes	Yes	DAAA02044420.1
*Bubalus bubalis*	Yes	Yes	Yes	AWWX01630119.1
Camelus dromedarius ^*^	Yes	Yes	Yes	LSZX01012659.1
*Camelus ferus*	Yes	Yes	Yes	AGVR01051296.1; AGVR01051297.1
*Canis lupus familiaris*	Yes	X	/	AAEX03011713.1
*Capra aegagrus*	Yes	Yes	Yes	CBYH010071014.1
*Capra hircus*	Yes	Yes	/	AJPT01162992.1; AJPT01162993.1
*Capreolus capreolus*	Yes	Yes	Yes	CCMK010092645.1; CCMK010104759.1
*Ceratotherium simum*	Yes	Yes	Yes	AKZM01017598.1
*Coelodonta antiquitatis*	Yes	Yes	Yes	MF871623
*Condylura cristata*	Yes	X	X	AJFV01047153.1
*Dicerorhinus sumatrensis*	Yes	Yes	Yes	MF871625
*Diceros bicornis*	Yes	Yes	Yes	MF871624
*Eidolon helvum*	Yes	Yes	/	AWHC01286101.1; AWHC01029981.1
*Eptesicus fuscus*	Yes	X	Yes	ALEH01005956.1
*Equus asinus*	Yes	Yes	Yes	JREZ01000001.1
*Equus caballus*	Yes	Yes	/	AAWR02018850.1; AAWR02018851.1
*Equus przewalskii*	Yes	Yes	Yes	ATBW01036321.1; ATBW01036322.1
*Erinaceus europaeus*	/	/	X	AMDU01193160.1; AMDU01193161.1; AMDU01193162.1
*Felis catus*	Yes	Yes	Yes	AANG02062919.1
Giraffa camelopardalis ^*^	Yes	Yes	X	LVKQ01071482.1
Hipposideros armiger ^*^	Yes	Yes	Yes	NW_017731683.1
*Leptonychotes weddellii*	Yes	Yes	Yes	APMU01115165.1; APMU01141180.1
*Lipotes vexillifer*	Yes	Yes	Yes	AUPI01000024.1
Lycaon pictus ^*^	Yes	X	/	LPRB01000019.1
Manis javanica ^*^	X	X	X	NW_016530114.1
*Manis pentadactyla*	X	X	X	JPTV01131901.1
*Megaderma lyra*	Yes	/	/	AWHB01167753.1; AWHB01348443.1; AWHB01348444.1
Miniopterus natalensis ^*^	Yes	X	Yes	NW_015504404.1
*Mustela putorius furo*	Yes	Yes	Yes	AGTQ01041845.1
*Myotis brandtii*	Yes	X	Yes	ANKR01273867.1; ANKR01273868.1
*Myotis davidii*	Yes	X	Yes	ALWT01125743.1
*Myotis lucifugus*	Yes	X	Yes	AAPE02001462.1
*Odobenus rosmarus*	Yes	Yes	Yes	ANOP01028105.1
Okapia johnstoni ^*^	Yes	Yes	Yes	LVCL010093660.1; LVCL010093662.1
*Orcinus orca*	X	X	X	ANOL02004931.1
*Ovis aries*	Yes	Yes	Yes	AMGL01037664.1; JN604985.1
Panthera pardus ^*^	Yes	Yes	Yes	NW_017619848.1
*Panthera tigris altaica*	Yes	Yes	Yes	ATCQ01112915.1
*Panthera uncia*	Yes	Yes	/	SRX273036
*Pantholops hodgsonii*	Yes	Yes	Yes	AGTT01188813.1
*Physeter macrocephalus*	X	Yes	Yes	AWZP01062081.1
*Pteropus alecto*	Yes	Yes	Yes	ALWS01011689.1
*Pteropus vampyrus*	Yes	Yes	Yes	ABRP02126915.1
*Rhinoceros unicornis*	Yes	Yes	Yes	MF871626
*Rhinolophus ferrumequinum*	Yes	Yes	Yes	AWHA01040305.1
Rhinolophus sinicus ^*^	Yes	Yes	Yes	NW_017738992.1
Rousettus aegyptiacus ^*^	Yes	Yes	Yes	NW_015494583.1
*Sorex araneus*	Yes	X	Yes	AALT02056093.1
*Sus cebrifrons*	Yes	/	/	ERX953604-ERX953626; ERX149172
*Sus scrofa*	Yes	X	X	LUXQ01106311.1
*Sus verrucosus*	Yes	/	/	ERX1054048-ERX1054067; ERX149174
*Tapirus indicus*	Yes	/	Yes	MF871627
*Tursiops truncatus*	X	X	X	ABRN02199412.1
*Ursus maritimus*	Yes	Yes	/	AVOR01014285.1; AVOR01014286.1
*Vicugna pacos*	Yes	Yes	Yes	ABRR02134987.1; ABRR02134989.1
**EUARCHONTOGLIRES**				
*Aotus nancymaae*	Yes	Yes	Yes	JYKP01215429.1
*Apodemus sylvaticus*	Yes	/	X	LIPJ01452544.1; LIPJ01184746.1; LIPJ01447868.1; LIPJ01014497.1
*Callithrix jacchus*	Yes	Yes	Yes	ACFV01002817.1
*Cavia aperea*	/	/	/	AVPZ01000778.1
*Cavia porcellus*	Yes	Yes	Yes	AAKN02011801.1
Cebus capuchinis ^*^	Yes	Yes	Yes	NW_016107319.1
*Cercocebus atys*	Yes	Yes	Yes	JZLG01060688.1
*Chinchilla lanigera*	Yes	Yes	Yes	AGCD01027651.1
*Chlorocebus sabaeus*	Yes	Yes	Yes	AQIB01017419.1
*Colobus angolensis*	Yes	Yes	Yes	JYKR01122839.1
*Cricetulus griseus*	Yes	Yes	X	AFTD01128393.1; AFTD01128394.1
*Daubentonia madagascariensis*	Yes	Yes	/	AGTM011584638.1; AGTM011584996.1; AGTM011708528.1; AGTM012010142.1; AGTM011594144.1
*Dipodomys ordii*	Yes	X	Yes	ABRO02057411.1
Ellobius lutescens ^*^	Yes	X	Yes	LOEQ01000193.1
Ellobius talpinus ^*^	Yes	X	Yes	LOJH01032235.1
*Eulemur flavifrons*	Yes	Yes	Yes	LGHW01000184.1
*Eulemur macaco*	Yes	Yes	Yes	LGHX01000184.1
*Fukomys damarensis*	Yes	Yes	Yes	AYUG01151056.1
*Galeopterus variegatus*	Yes	Yes	/	JMZW01045215.1; JMZW01045216.1
*Gorilla gorilla gorilla*	Yes	Yes	Yes	NW_004002547.1
*Heterocephalus glaber*	Yes	Yes	Yes	AFSB01162372.1; AFSB01162373.1
*Homo sapiens*	Yes	Yes	Yes	NG_012139.1
*Jaculus jaculus*	Yes	Yes	Yes	AKZC01091543.1
*Macaca fascicularis*	Yes	Yes	Yes	CAEC01514737.1
*Macaca mulatta*	Yes	Yes	Yes	AANU01271750.1
*Macaca nemestrina*	Yes	Yes	Yes	JZLF01028562.1
*Mandrillus leucophaeus*	Yes	Yes	Yes	JYKQ01107154.1; JYKQ01107155.1
*Marmota marmota*	Yes	Yes	Yes	CZRN01000015.1
*Mesocricetus auratus*	Yes	X	Yes	APMT01116524.1; NM_001281332.1
*Microcebus murinus*	Yes	Yes	Yes	ABDC01082367.1
*Microtus agrestis*	Yes	X	Yes	LIQJ01004042.1
*Microtus ochrogaster*	Yes	X	X	AHZW01157105.1; AHZW01157106.1
*Mus musculus*	Yes	X	X	CAAA01024310.1
Mus spretus ^*^	Yes	X	X	LVXV01001867.1
*Myodes glareolus*	Yes	/	/	LIPI01003929.1
*Nannospalax galili*	Yes	Yes	X	AXCS01128925.1
*Nasalis larvatus*	Yes	Yes	Yes	JMHX01319533.1
Neotoma lepida ^*^	/	/	X	LZPO01075894.1
*Nomascus leucogenys*	Yes	Yes	Yes	ADFV01177960.1
*Ochotona princeps*	Yes	X	X	ALIT01060999.1
*Octodon degus*	Yes	Yes	Yes	AJSA01193669.1; AJSA01193670.1; AJSA01193671.1
*Oryctolagus cuniculus*	Yes	Yes	Yes	AAGW02045633.1
*Otolemur garnettii*	Yes	Yes	X	AAQR03074138.1
*Pan paniscus*	Yes	Yes	Yes	AJFE01070904.1
*Pan troglodytes*	Yes	Yes	/	AACZ03032212.1; AACZ03032213.1
*Papio anubis*	Yes	Yes	Yes	AHZZ01043343.1
*Peromyscus maniculatus*	Yes	X	X	AYHN01134223.1
*Pongo abelii*	Yes	Yes	Yes	ABGA01062109.1
*Propithecus coquereli*	Yes	Yes	Yes	JZKE01017273.1
*Rattus norvegicus*	Yes	X	X	AAHX01097782.1
Rhinopithecus bieti ^*^	Yes	Yes	Yes	NW_016805762.1
*Rhinopithecus roxellana*	Yes	Yes	Yes	JABR01098768.1
*Saimiri boliviensis*	Yes	Yes	Yes	AGCE01051213.1
*Spermophilus tridecemlineatus*	Yes	Yes	Yes	AGTP01049378.1
*Tarsius syrichta*	Yes	Yes	/	ABRT02355486.1
*Tupaia belangeri chinensis*	Yes	Yes	Yes	ALAR01031045.1

Xs = absent, / = inconclusive due to insufficient data,

* *= 16 species with recently published genome projects since the Gaudry et al. (2017) publication. Accession numbers are also provided for contigs and SRA projects*.

For publically available genomes, *UCP1* regulatory sequences were acquired using genome-mining techniques of sequences available on the National Center for Biotechnology Information web server. *UCP1*-containing contigs were first acquired by performing nucleotide BLAST searches employing the “discontinuous megablast” option against whole genome shotgun (WGS) contigs of mammalian genome projects using human *UCP1* CDS (NM_021833.4) as a query. If the contigs did not extend ~5 kb upstream of the *UCP1* transcriptional start site to include the enhancer box, an additional nucleotide BLAST was performed using the human *UCP1* enhancer sequence as a query. For several species with genome projects that have not yet been fully assembled (e.g., *Sus cebifrons, Sus verrucosus, Elephas maximus, Mammuthus primigenius, Balaena mysticetus, Balaenoptera physalus, Mylodon darwinii, Panthera unica*), short read archive (SRA) BLASTs were performed in order to obtain the *UCP1* regulatory elements. Contigs from top BLAST hits were then imported into Sequencher v5.1 (Gene Codes Corporation; Ann Arbor, Michigan, USA) and the exons and regulatory regions annotated by aligning orthologous human *UCP1* sequences (exons 1–6 and enhancer), initially at a 85% minimum match percentage. If the sequences were too divergent to assemble at that stringency, the minimum match percentage was progressively decreased to 60% or until the sequences successfully assembled. *UCP1* coding regions for the 16 species not included in the Gaudry et al. (2017) study were also examined for the presence of inactivating (e.g., splice site, frameshift, and non-sense) mutations.

The PRR proposed by Shore et al. (2012) was generally less conserved than the enhancer, often with large insertions or deletions, therefore the same annotation methods described above could not be effectively applied to this region. Instead, dot plots were performed in Geneious R9.1 (Biomatters Ltd.) which uses the EMBOSS 6.5.7 dotmatcher tool to compare sequence identities of the human PRR vs. the upstream sequence of other mammalian species using a window size of 25, a threshold of 45, and the high sensitivity setting with a probabilistic scoring matrix. The PRR was determined to be present if a conserved region >100 bp relative to the human sequence was discernible from the dot plots. The boundaries of the PRRs were estimated using the dot plot and annotated. The PRRs of species listed in Table 2 were then screened in rVista 2.0 (Loots and Ovcharenko, 2004) for the presence of putative transcription factor binding motifs [DR1, DR3, DR4, CEBP (CCAAT-enhancer-binding proteins), CREB, and PPAR] shared with humans, as performed by Shore et al. (2012). Insertions larger than 100 bp relative to the human PRR were removed prior to screening in rVista using the vertebrate TRANSFAC professional V10.2 library with the “matrix similarity optimized for function” setting.

**Table 2:** Possible transcription factor binding motifs within the PRR of selected species screened using rVista 2.0.

**Species**	**Motif**	**Position**	**Sequence**
*Homo sapiens*	CREB	24+	catggCATCAgttc
	DR3	227−	cagaGGTTCACTAGAGTCaac
	DR4	230−	agGTTCACTAGAGTCAa
*Marmota marmota*	PPAR_DR1	50−	tGGTCAAAGGACt
	DR4	326−	tgGGTCCCTTAAGGTCa
	DR1	393−	TGACACTTATCCc
*Oryctolagus cuniculus*	CREB	373−	ccTAACATCAcc
	CEBP	519−	gcTCCATTGCCTAACTCt
	PPAR_DR1	592+	tGGCCCTTGGCCc
	PPAR_DR1	601+	gCCCCTTTGTCCc
*Camelus ferus*	CEBP	271−	taTACATTTGGGCATACt
	CEBP	503−	tgTTCCTTTCCTAATTGt
	CREB	636−	tgtCATCAcct
*Bos taurus*	CREB	149+	CGTCAg
	CEBP	240−	taTGCATTATAACAAACa
	CEBP	471−	tgTTTCTTTCCTAATTTg
	PPAR_DR1	487+	tGACCTTTGATAa
	PPAR_DR1	542+	tGACCCTTGACCc
*Giraffa camelopardalis*	CREB	150+	CGTCAg
	CREB	476−	tgTTTCTTTCCTAATTTg
	PPAR_DR1	492+	tGACCTTTGATAa
	PPAR_DR1	547+	tGACCCTTGACCc
*Balaenoptera acutorostrata*	DR1	96+	aGGGGAAGGGACA
	CEBP	518−	taTTTCTTTCCTAACTTt
	PPAR_DR1	587+	tGGCCCTTGACCc
	DR1	587−	TGGCCCTTGACCc
	DR1	594−	TGACCCCTTTCCc
*Lipotes vexillifer*	DR3	291+	accGAACATTCTCAATCtgct
	CEBP	509−	taTTTCTTTCCTAACTTt
	PPAR_DR1	580+	tGGCCCTTGACCc
	DR1	587−	TGACCCCTTTCCc
*Ceratotherium simum*	DR1	108+	aGGGGAAGGGACA
	DR4	246−	agGATCACTAGAGTTAg
	CEBP	284−	taTACATTTAGTCATACt
	DR3	304+	accGAACATTCTCAATCtctg
	DR4	425+	tGTCCTCTTTTGACAtt
	PPAR_DR1	453+	tCACACTTGACCc
*Equus przewalskii*	CEBP	9+	cTTTCACAAtcc
	CREB	36−	caTAGCGTCAgt
	CREB	41+	CGTCAg
	DR4	234−	agGTTCACTAGAGTTAg
	PPAR_DR1	537+	tTACCTTTGACCa
	DR1	592−	TGGTCCTTGACCc
	CREB	667+	ttGCTGACTccc
*Equus caballus*	DR4	224−	agGTTCACTAGAGTTAg
	PPAR_DR1	524+	tTACCTTTGACCa
	DR1	579−	TGGTCCTTGACCc
	CREB	654+	ttGCTGACTccc
*Pteroupus vampyrus*	CREB	37+	catagCATCAgctc
	DR4	408+	tGTCCTCTTTTGACAtt
	PPAR_DR1	575+	tGGCCCTTGACCc
	DR1	582−	TGACCCCTTTCCt
*Ailuropoda melanoleuca*	DR1	85+	aGGGGAAGGGACA
	CREB	505+	ttGATGAGGccc
	DR1	554−	TGGCCCATGACCc
	PPAR_DR1	561+	tGACCCTTTGCCt
	CREB	628+	ttGCTGACTccc
*Odobenus rosmarus*	DR1	92+	aGGGGAAGGGACA
	DR4	406+	tGTCCTCTTTTGACAtt
	DR1	567−	TGGCCCATGACCc
	PPAR_DR1	574+	tGACCCTTTTCCt
	CREB	670+	ttGCTGACTccc
*Panthera pardus*	DR4	240+	tGTCCTCTTTTGACAca
*Leptonychotes weddellii*	DR1	90+	aGGGGAAGGGACA
	DR4	403+	tGTCCTCTTTTGACAtt
	DR1	564−	TGGCCCATGACCc
	PPAR_DR1	571+	tGACCCTTTTCCt
	CREB	671+	ttGCTGACTccc
*Procavia capensis*	CREB	59−	ccTAACATCAcc
	DR1	273−	TGGTCCTTGACCt
	CREB	278+	cttgaCCTCAttgc
	CREB	280+	TGACCTca
*Loxodonta africana*	CREB	32+	acataCATCAgctc
	CREB	347−	caTAACATCAcc
	CREB	424−	tTGACG
	PPAR_DR1	566+	tGGCCCTTGACCc
*Trichechus manatus*	CREB	140−	tgAGGTCA
	CREB	369−	taaCATCACCaa
	PPAR_DR1	587+	tGGCCCTTGACCc
*Echinops telfairi*	PPAR_DR1	189−	gGGTCAAGGATCa
	CREB	326−	ccTGACATCAct

*Duplicates sites were removed. Position is indicated relative to the start of the PRR sequence and the strand is indicated with + or − symbols*.

Basal promoter regions were identified by performing alignments of 600 bp upstream of the ATG start codon for each species with available sequence data. The rat and mouse upstream sequences contain several putative promoter motifs (e.g., TATA box, CCAAT site, CRE-4, and GCCCCT sites) and thus were used as reference sequences. CpG islands within the 5′ region of *UCP1* were identified using the EMBOSS CpGplot tool (http://www.ebi.ac.uk/Tools/seqstats/emboss_cpgplot/). Kiskinis et al. (2007) noted that the *UCP1* CpG island occurs immediately upstream of the *UCP1* open reading frame but may also extend into exon 1, therefore, 1 kb upstream of exon 2 was screened for the presence of CpG islands. EMBOSS CpGplot positively identifies CpG islands if a sequence >200 bp contains an observed/expected ratio of CpGs exceeding 0.6, with a GC content >50%, meeting the criteria proposed by Gardiner-Garden and Frommer (1987). The default window size of 100 bp was used for these runs.

The *UCP1* genes of non-eutherian mammals were also examined for the presence or absence of regulatory elements. Contigs of the Tasmanian devil (*Sarcophilus harrisii*) and Tammar wallaby (*Macropus eugenii*) were too short to encompass a potential enhancer occurring ~5 kb upstream of the transcriptional start site. However, contigs of the platypus (*Ornithorhynchus anatinus*) and gray short-tailed opossum were sufficiently long to create dot plots of the upstream sequence in order to screen for homologous regulatory elements occurring in the human. Some eutherian species displayed inactivated *UCP1* genes with deletions of whole exons (e.g., Chinese pangolin; *Manis pentacatyla*, Javan pangolin; *Manis javanica*, nine-banded armadillo; *Dasypus novemcinctus*), or deletion of the entire gene (killer whale and bottlenose dolphin). The annotation techniques described above did not reveal the presence of a *UCP1* enhancer in these species; thus, sequence identity comparisons against human *UCP1* were performed using Easyfig 2.1 (Sullivan et al., 2011). This analysis was also performed for the rat and cow (*Bos taurus*) since these were species are known to display *UCP1* enhancers while the cow also contains a PRR region (Shore et al., 2012).

Finally, regions containing enhancer and basal promoter sequences for each species were imported into Geneious 9.1 and multispecies nucleotide alignments were generated using the MUSCLE alignment tool (Edgar, 2004) with default settings. A consensus eutherian sequence representing the simple majority (>50%) was generated from this dataset based only on species for which the *UCP1* gene is intact (i.e., species with documented *UCP1* pseudogenes (Gaudry et al., 2017) were not included in the consensus calculations). For some eutherian species, pairwise alignments were also created against the human enhancer to obtain the percent sequence identity values. Conserved motifs and putative transcription factor binding sites were annotated. Recognized transcription factor binding motifs within the *UCP1* enhancer (illustrated in Figure 2) were examined by eye in each eutherian species and scrutinized for mutations that potentially affect DNA-protein interactions based on previous site directed mutagenesis studies. Additionally, the consensus enhancer region sequence (see above), together of those of seven species spanning the three mammalian superorders for which *UCP1* is intact, were screened for the presence of all vertebrate transcription factors in the TRANSFAC professional V10.2 library using rVista with the “matrix similarity optimized for function” setting.

### Phylogenetic trees

To generate a combined *UCP1, UCP2*, and *UCP3* coding sequence phylogenetic tree, the data set of Gaudry et al. (2017) was updated to include coding sequences of the 16 additional species with recently published genomes (Table 1). The resulting 448 *UCP* genes were aligned using MUSCLE (Edgar, 2004), and a maximum likelihood tree constructed using RAxML (Randomized Axelerated Maximum likelihood) version 7.2.8 (Stamatakis, 2006) with the “GTR Gamma” nucleotide model and “rapid bootstrapping and search for best scoring tree” setting. The program was performed for 500 bootstrap replicates.

In order to trace the evolutionary gain and loss of *UCP1* transcriptional regulatory elements, we also constructed a 41-gene species tree for the 139 mammals included in this study following the methods of Gaudry et al. (2017). Briefly, this data set included coding and non-coding sequences from 30 nuclear (*A2AB, ADRB2, APP, ATP7A, ADORA3, APOB, BCHE, BDNF, BMI1, BRCA1, BRCA2, CHRNA1, CMYC, CNR1, CREM, DMP1, ENAM, EDG1, FBN1, GHR, IRBP, MC1R, PLCB4, PNOC, RAG1, RAG2, SWS1, TTN, TYR1, VWF*) and 11 mitochondrial loci (*12S rRNA, 16S rRNA, CYTB, COI, COII, COIII, ND1, ND2, ND3, ND4, ND5*). A 50,911 bp concatenated supermatrix was aligned in MUSCLE. The supermatrix was divided into 32 partitions (see Supplementary Materials). Each nuclear gene was assigned an individual partition, while *12S rRNA* and *16S rRNA* were combined to create one partition, and the nine remaining mitochondrial genes were also combined into a single partition. An independent GTR Gamma model was estimated for of these partitions and a maximum likelihood tree was generated in RAxML 7.2.8 using the same settings described above with 100 bootstrap replicates.

## Results

### *UCP1* coding sequences

All of the 16 newly acquired *UCP1* CDSs were intact with the exception of the Javan pangolin, which displays the same mutations as the Chinese pangolin pseudogene (i.e., frameshift, splice site and non-sense mutations, deletion of exons 1 and 2) documented by Gaudry et al. (2017). Similarly, the 12 bp deletion that calls into question the functionality of the Bactrian camel *UCP1* gene (Gaudry et al., 2017) is also present in the dromedary camel (*Camelus dromedarius*). Conversely, the *UCP1* CDS of the giraffe (*Giraffa camelopardalis*) is intact, despite its large body size.

**Figure 3:**
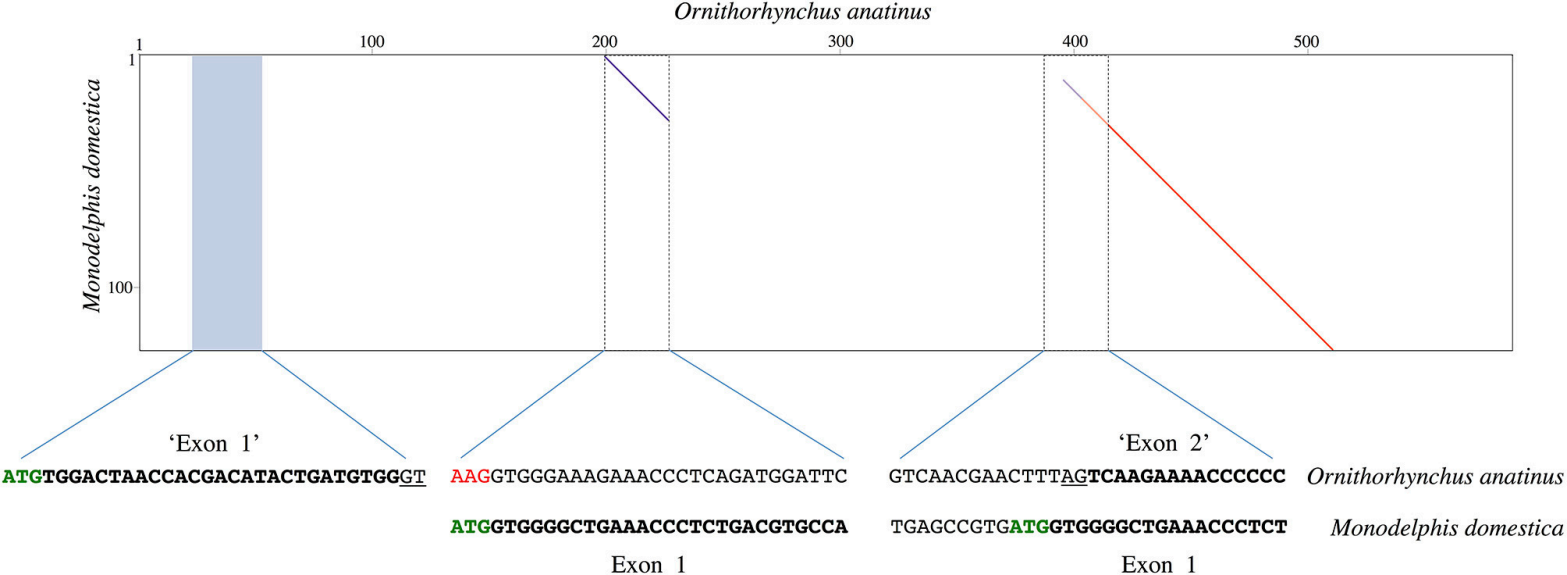
Dot plot comparison of the gray short-tailed opossum *UCP1* exon 1 vs. a section of the platypus *UCP1* gene occurring between *TBC1D9* and *ELMOD2* (accession number: NW_001794248.1). Sequence alignments of the platypus (**top**) and gray-short tailed opossum (**bottom**) are provided with the potential coding sequences indicated in bold; putative splice sites are underlined. Note that two regions within the platypus clearly display homology to the opossum exon 1 (199–226 and 400–520), suggesting the presence of a 186 bp insertion in the platypus exon 1 sequence. The blue shaded area represents the region where an automated predictor program, which created a seven exon *UCP1* gene for the platypus, placed a 30 bp “exon 1” in order to obtain an open reading frame free from premature stop codons (accession number: XM_001512650), though this region shares no homology with exon 1 of the opossum. The original platypus start codon also appears to be mutated to AAG (red font), with the predicted platypus “exon 2” occurring 6 bp downstream of the “ATG” start site in the opossum. Note that these differences between the two species likely arise from a misassembly error in the platypus (see text for details).

The predicted platypus *UCP1* CDS available on GenBank (accession number: XM_001512650) is unique in that it creates a hypothetical open reading frame composed of seven exons; the usual 126 bp exon 1 is divided into two separate exons of 30 and 120 bp in length. The placement of these putative exons are displayed in a dot plot comparison with the 5′ region of the gray short-tailed opossum *UCP1* locus (Figure 3). Notably, two separate regions within the platypus read display homology to the opossum *UCP1* exon 1 sequence, revealing what appears to be a 186 bp insertion in the platypus exon 1 sequence. The original platypus start codon also appears to be mutated to “AAG” thus translocating the predicted 30 bp ‘exon 1′ of the platypus 176 bp upstream of the gray short-tailed opossum start codon (Figure 3). By contrast, BLAST searches of platypus RNA sequencing projects (SRX182802, SRX17144, SRX17145, SRX081892, SRX081881, SRX081882, SRX328084, SRX328085, SRX081887-SRX081890) reveal an intact *UCP1* mRNA sequence (Supplemental File 5) that differs from the predicted coding sequence. Briefly, the platypus mRNA coding sequence indicates that the predicted 30 bp “exon 1” coding sequence is not translated, that there is no insertion in exon 1 of the platypus, and that the ATG start codon found in other mammals is indeed intact at the expected position (i.e., there is a misassembly error in the predicted GenBank sequence).

### *UCP1* basal promoter

**Figure 4:**
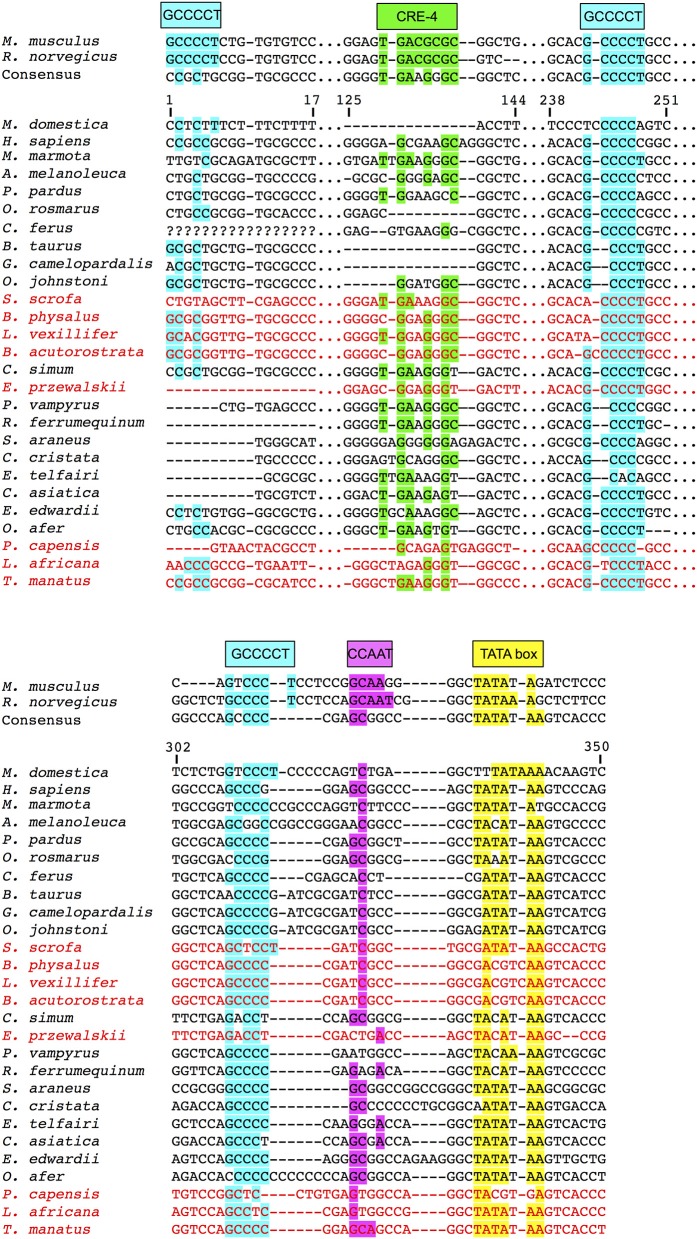
*UCP1* basal promoter elements alignment for select mammalian species with putative protein binding motifs indicated. Highlighted sites indicate shared nucleotides to the species in which the motif was first described (mouse or rat) and the typical TATA box (5′-TATAAAA-3′) sequence (Xu et al., 1991). The consensus sequence represents the simple majority based on species for which the *UCP1* gene is intact. Species with documented *UCP1* pseudogenes (Gaudry et al., 2017) are denoted in red font and were not included in the consensus calculations.

An alignment of the basal *UCP1* promoter for representative species is displayed in Figure 4. Notably, the most upstream GCCCCT motif (nucleotides 1–6 of the promoter alignment; Figure 4) described in the rat by Yubero et al. (1994) is not present in any non-murid species. While the CRE-4 consensus sequence (5′-TGAAGGGC-3′) is similar to that described by Kozak et al. (1994) in mice (5′-TGACGCGC-3′), this site does differ substantially in many species (e.g., common shrew [*Sorex araneus*], human, etc.) and is absent in the gray short-tailed opossum, walrus, cow, and giraffe (Figure 4). The second and third GCCCCT sites, respectively occurring at 242–248 and 308–315 of the alignment, are relatively well conserved (Figure 4). By contrast, the putative CCAAT site in the rat (Bouillaud et al., 1988) is highly variable in other mammals. The TATA box described by Bouillaud et al. (1988) is intact in the majority of species including all marsupials where it occurs as a 5′-TATAARR-3′ sequence 260–280 upstream of the ATG start codon of exon 1. While a 5′-TATAAGG-3′ sequence is found ~200 bp upstream of the platypus *UCP1* coding sequence, the validity of this site is uncertain due to a misassembly in this region of the GenBank sequence (see above). Interestingly, the walrus motif contains a T → A mutation causing a 5′-TAAATAA-3′ sequence, while the panda, white rhinoceros, horse, and bats share a 5′-TACAWAA-3′ sequence. Among species that possess pseudogenized *UCP1* genes, an intact TATA box still remains ~290 bp upstream of the African elephant (*L. africana*) and manatee (*T. manatus*) coding sequence while the closely related Cape rock hyrax (*Procavia capensis*) deviates from the consensus (5′-TACGTGA-3′). Similarly, the pig retains a TATA box identical to that of the cow, camel, and giraffe (5′-GATATAA-3′), though a number of mutations in cetaceans have resulted in a sequence (5′-GACGTCAA-3′) that is virtually unrecognizable as a TATA box (Figure 4).

### CpG island

**Figure 5:**
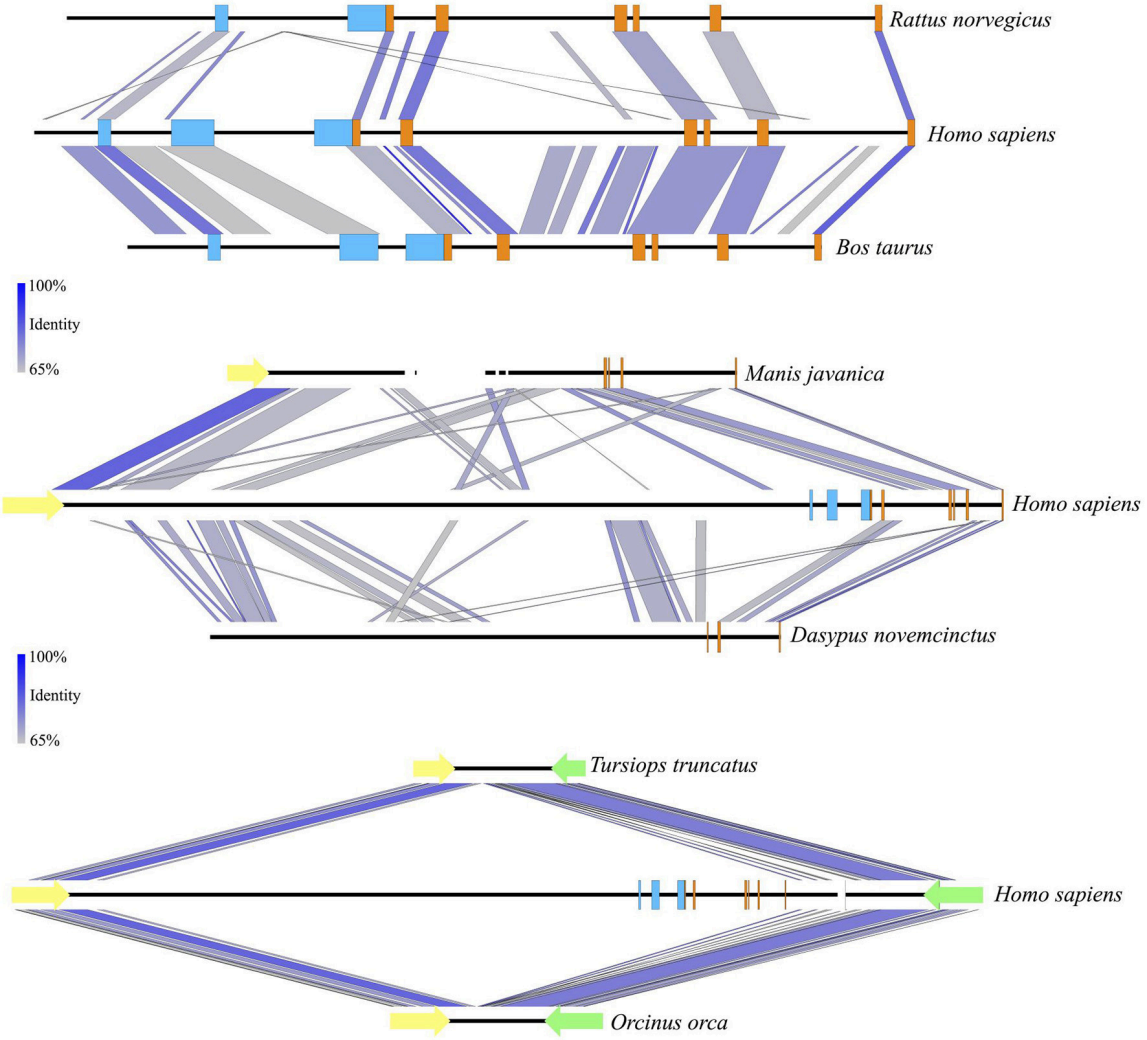
Sequence identity comparisons of the *UCP1* genes of the rat, cow, pangolin, armadillo, bottlenose dolphin, and killer whale vs. the human. All DNA sequences are shown 5′ (**left**) to 3′ (**right**). *UCP1* exons 1–6 are denoted with orange rectangles while *UCP1* upstream transcriptional regulatory elements are denoted in light blue (enhancer box, putative regulatory region, CpG island; from left to right). Gaps in sequence coverage are represented by white rectangles. Notably, the putative regulatory region is absent in the rat, but conserved in the cow. Upstream regulatory elements also appear to have been deleted in the Javan pangolin and armadillo, which have deletions of *UCP1* exons 1–2, and 3–5, respectively. Deletion of the entire *UCP1* gene between *TBC1D9* (yellow arrows) and *ELMOD2* (green arrows) has occurred in bottlenose dolphin and killer whale ~8–15 MYA (Gaudry et al., 2017) and included the upstream regulatory elements. Sequence identity percentage is represented with a color scale.

CpG islands meeting the criteria of Gardiner-Garden and Frommer (1987) were not detected in the monotreme or marsupial assemblies. Conversely, a CpG island within or immediately upstream of exon 1 was identified in 91 of 113 eutherian species with available sequence coverage for this region (Table 1). The presence of the CpG island was found to vary extensively among small-bodied species as it was detected in the common shrew, but is absent from the European hedgehog (*Erinaceus europaeus*) and star-nosed mole (*Condylura cristata*; Table 1). Many rodent species (e.g., mouse, rat), known to express functional BAT, also lack a CpG island (Table 1). Similarly, among the four afroinsectiphilians examined, a CpG island was only identified in the lesser hedgehog tenrec (containing 39 CpG dinucleotides), despite a relatively high number of CpG sites (37–41) located between 600 bp upstream and 200 bp downstream of the start codon in the other three species. Conversely, CpG islands were identified in closely related paenungulates (elephants, sirenians, and hyraxes), which have >50 CpG dinucleotides in the same region, and armadillos—despite both of these groups having a non-functional *UCP1*. Among artiodactyls, CpG islands were detected in camels, the okapi (*Okapia johnstoni*), and all whale *UCP1* pseudogenes (except for the killer whale and bottlenose dolphin for which the entire gene is deleted; Figure 5), but not the giraffe or the pig (*Sus scrofa*). This element is also missing in the pangolin pseudogenes, which is likely due to deletion of a portion of the gene upstream of exon 3 (Figure 5).

### Putative regulatory region (PRR)

**Figure 6:**
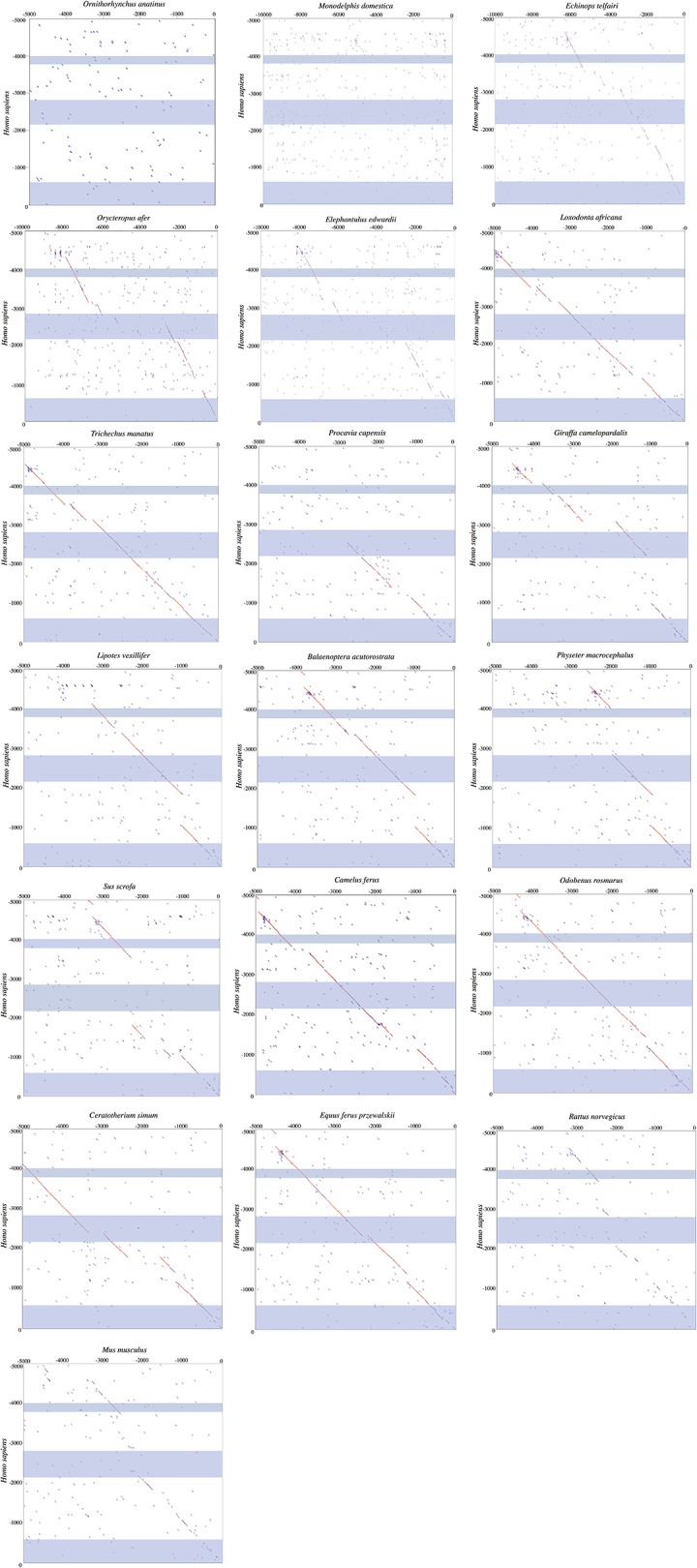
Dot plots of the 5,000 or 10,000 bp upstream of *UCP1* exon 1 of select mammalian species compared to the upstream sequence of humans. Blue shading represents the *UCP1* enhancer (~ −4,000 to −3,800 in human), putative regulatory region (~ −2,700 to −2,500 in human), and promoter/CpG island (−600 to 0 in human), in that order, from top to bottom.

A distinct PRR was found to be present in 97 of the 125 eutherian mammals examined for which sequence is available (Table 1), though this element was not observed in the platypus or gray short-tailed opossum (Figure 6). PRRs were observed from all afrotherians, but not the armadillo, a xenarthran (Table 1), though insertions within this region are prevalent in the elephant shrew, lesser hedgehog tenrec, and aardvark (Figure 6). By contrast, the dot plots of the elephant and manatee—for which *UCP1* is pseudogenized—reveal a high conservation of the PRR with virtually no indels, though only the 3′ half of the PRR is present in the hyrax (Figure 6). As seen for the cow (Figure 5), giraffe, camel, and several whales (Figure 6), the PRR is conserved among most artiodactyls, but is missing in the pig *UCP1* pseudogene (Figure 6) and deleted in the bottlenose dolphin, killer whale, and Javan pangolin (Figure 5). A PRR is also absent in several species known to express functional BAT, including the shrew and star-nosed mole, several bats (*Myotis* spp. and *Eptesicus fuscus*, etc.), and many rodents (Table 1), including the mouse and rat (Figures 5, 6). Similarly, both *Canis familiaris* and *Lycaon pictus* lack a PRR, despite this feature being present in all other carnivores (Table 1). The transcription factor binding sites identified within PRRs of selected species using rVista 2.0 are listed in Table 2. PPAR, DR1, DR3, DR4, CREB, and CEBP sites are relatively common within this region in species with and without a functional *UCP1* locus.

### *UCP1* enhancer

*UCP1* enhancer sequences were retrieved for 121 eutherian species (Table 1). Enhancer boxes were typically found within 5 kb upstream of exon 1, however, for some members of the afroinsectiphilia (i.e., aardvark and elephant shrew), the enhancer occurs at ~ –7.5 kb (Figure 6). Dot plots of the upstream regions of the platypus and the gray short-tailed opossum reveal no evidence for a *UCP1* enhancer (Figure 6), suggesting it is absent within both monotremes and marsupials.

Contrary to the findings of Shore et al. (2012), who noted the absence of an enhancer in the upstream region of the common marmoset (*Callithrix jacchus*), American pika (*Ochotona princeps*), thirteen-lined ground squirrel (*Spermophilus tridecemlineatus*), common shrew, and European hedgehog, we identified this element in each of these species except the hedgehog. The contig encompassing hedgehog *UCP1* CDS (accession number: AMUD01193160.1), however, only extends 1126 bp upstream of exon 1 and BLAST searches failed to provide hits of a *UCP1* enhancer located on other contigs, thus its presence or absence from the genome remains inconclusive. Similarly, low sequencing coverage likely explains the apparent lack of a *UCP1* enhancer in the zebu (*Bos indicus*), Brazilian guinea pig (*Cavia apera*), and desert woodrat (*Neotoma lepida*), as enhancers have been recovered from their close phylogenetic relatives (Table 1).

**Figure 7:**
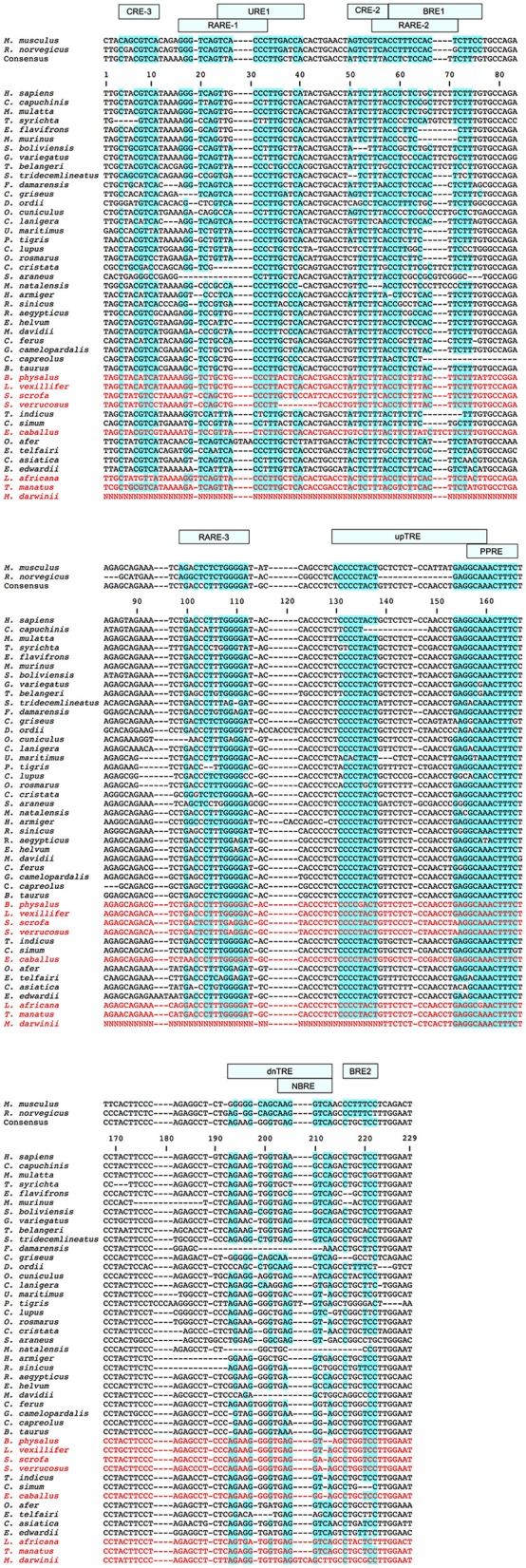
*UCP1* enhancer alignment for select eutherian species. Sequences highlighted in blue denote the degree of conservation relative to transcription factor binding sites first described in mice or rats (see also Figure 2). The consensus sequence represents the simple majority based on species for which the *UCP1* gene is intact. Species with documented *UCP1* pseudogenes (Gaudry et al., 2017) are denoted in red font and were not included in the consensus calculations.

The enhancer is highly conserved in large-bodied species with intact *UCP1* loci (i.e., rhinoceroses, camels, giraffe, and pinnipeds) as well as several species with *UCP1* pseudogenes (e.g., elephantids, sirenians, suids, equids, and some cetaceans; Table 1). However, seven species lack both a *UCP1* enhancer and an intact *UCP1*. For instance, the entire *UCP1* gene including the enhancer has been deleted in the killer whale and bottlenose dolphin (Figure 5). The enhancer has also been deleted in the sperm whale (*Physeter macrocephalus*; Figure 6), yet it remains present in the baiji (*Lipotes vexillifer*) and all baleen whales, indicating an independent loss in both the sperm whale and delphinids. The dot plots also fail to provide evidence for an *UCP1* enhancer in the Cape rock hyrax, though this element is present in other paenungulates for which this gene is also pseudogenized (Figure 6). Sequence identity comparisons also suggest the enhancer is lost in pangolins and the nine-banded armadillo (Figure 5 and Table 1). Interestingly, BLAST searches failed to identify this regulator in the WGS contigs or SRA of the two-toed sloth (*Choleopus hoffmanni*), although partial coverage was recovered for the extinct giant ground sloth (*M. darwinii*) from a pair of SRA reads (Figure 7).

**Figure 8:**
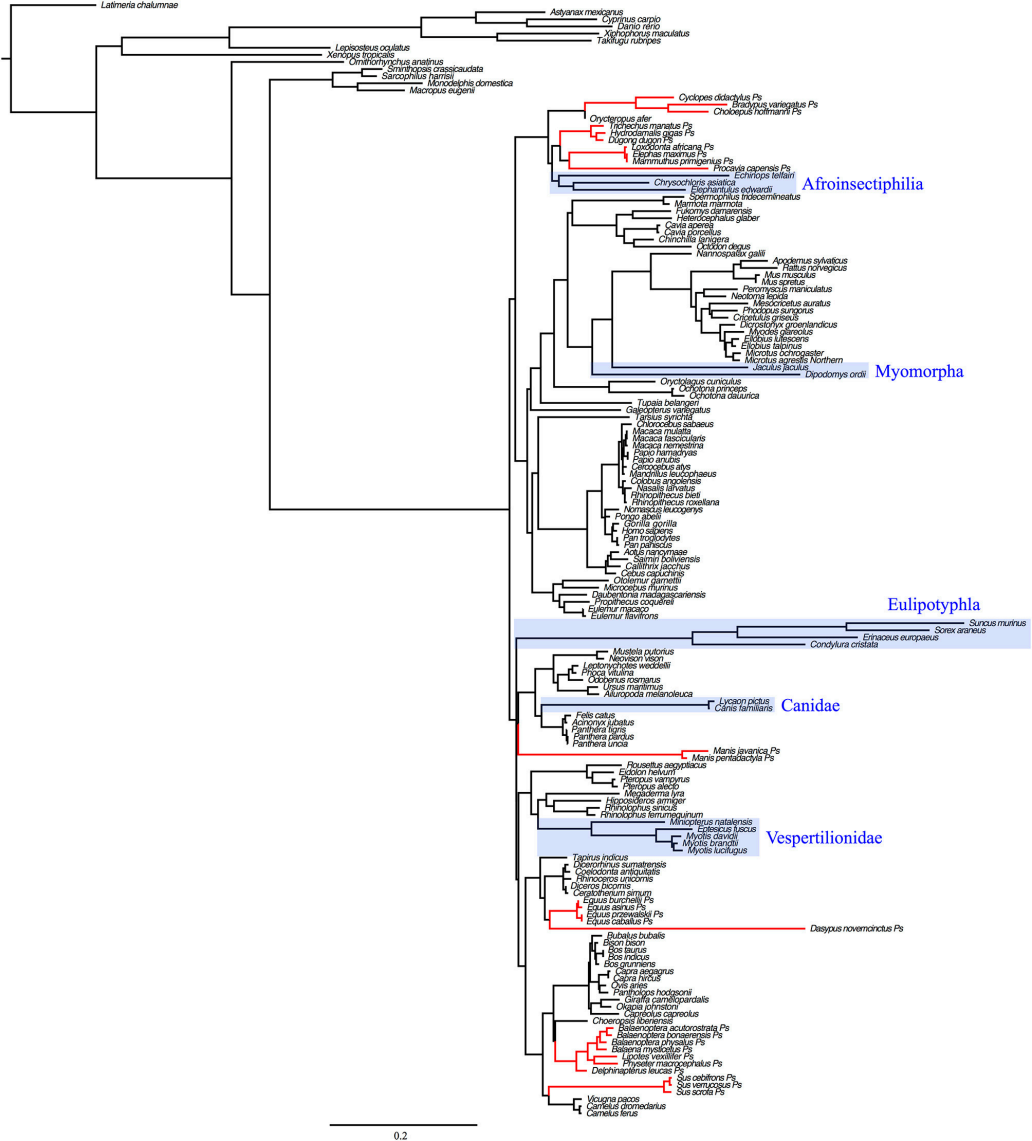
Maximum likelihood *UCP1* coding sequence gene tree illustrating substitution rates in several eutherian lineages (eulipotyphlans, canids, afroinsectiphilians, vesper bats, myomorph rodents; boxed in blue) that are comparable or higher than lineages with *UCP1* pseudogenes (denoted in red). Branch lengths represent the number of nucleotide substitutions per site.

Dot plots of the murid (rat and mouse) upstream sequence (Figure 6) illustrate marked divergence from humans with the exception of a small region encompassing the *UCP1* enhancer. By contrast, the upstream sequence of many laurasiatherians, and even paenungulates lacking an intact *UCP1* (e.g., elephants and manatees) is surprisingly similar to that of humans (Figure 6). In fact, pairwise sequence comparisons of these enhancers vs. that of the human reveal that this region is more highly conserved (>80%) in large-bodied species that both possess and lack an intact *UCP1* than the mouse (74%) and rat (69%) *UCP1* (data not show), despite the latter sharing a more recent common ancestor with humans. This pattern is mirrored in the *UCP1* gene tree (Figure 8) as many small-bodied lineages (i.e., afroinsectiphlians, myomorph rodents, vesper bats, and most notably, eulipotyphlans) display long branch lengths indicative of high rates of molecular evolution that are comparable to those of many species with *UCP1* pseudogenes (e.g., pangolins, pigs, armadillo, and hyrax). Canines are also worth noting, as their branch is highly elongated compared to other carnivores. By contrast, short branches found for most large-bodied species, even among those with non-functional *UCP1* (e.g., paenungulates, cetaceans, and equids), reflect low nucleotide substitution rates.

Enhancer region alignments revealed a number of marked differences within transcription factor binding motifs among species (Figure 7). For instance, while the CRE-3 site contains a set of core nucleotides (5′-CGTCA-3′) that are highly conserved in most eutherians, mutations to one or two nucleotides within this region are observed in a number of species (e.g., *C. cristata, Dipodomys ordi, Cricetulus griseus*), while the 5′ portion of this site appears to be deleted in the Philippine tarsier (*Tarsius syrichta*). Notably, the CRE-3 motif was detected in each species for which the enhancer was screened in rVista except for *C. cristata* (Table S1). Various mutations to this motif are also found in species with a pseudogenized *UCP1* (e.g., elephants, pigs, whales, and horses; Figure 7). The RARE-1 site is especially conserved in the section that overlaps with the URE1 motif, where the consensus sequence (5′-TTACCCTTGCTCA-3′) closely resembles the mouse URE1 site proposed by Sears et al. (1996). However, mutations at sites (e.g., nucleotide positions 32–33 of the alignment in Figure 7) shown to block transcription binding in mice (Sears et al., 1996) are observed in several species with intact *UCP1* (e.g., rabbit; *Oryctolagus cunculus*, Philippine tarsier; *T. syrichta*, white rhino; *C. simum*, and tapir; *T. indicus*). The aardvark displays a 4 bp insertion occurring within the URE1 that results in a single nucleotide (C → A) substitution to this motif. Notably, among species lacking a functional *UCP1*, the Javan warty pig (*S. verrucosus*) exhibits a marked disruption to the URE1 site.

The CRE-2 motif is well conserved among most eutherians, however, the consensus eutherian sequence (5′-ATTCTTTA-3′; Figure 7) is a poor match to the mouse 5′-AGTCGTCA-3′ sequence (Kozak et al., 1994). Indeed, of seven species for which the enhancer region was screened using rVista, this site was identified as a cAMP response element only within the mouse (Table S1). Notably, several species with an intact *UCP1* display deletions within the CRE-2 motif (e.g., black capped squirrel monkey; *Simiri boliviensis*, thirteen-lined ground squirrel; *S. tridecemlineatus*, and natal long-fingered bat; *Miniopterus natalensis*). Similarly, the two TTCC motifs described for the mouse BRE-1 site (Kozak et al., 1994) are not found in any non-murid eutherians. This region, however, is TC-rich in nearly all species with a single convergent TTCC site found in the dog and natal long-fingered bat (Figure 7). In contrast, the AT-richness of the BRE-1/RARE-2 region is substantially increased in horses, whales, and pigs—all of which lack a functional UCP1—relative to species with an intact gene.

The RARE-3 site consensus sequence (5′-TGACCCTTTGGGGAT-3′; Figure 7) is strongly conserved among eutherians with the exception of a 2-bp deletion in the tiger (*Panthera tigris*). The PPRE motif predicted by Jastroch et al. (2008) is also a highly conserved element within the *UCP1* enhancer, with a consensus sequence of 5′-GCAAACTTTC-3′. Of note, a PPARG (or PPARγ) site with a consensus sequence of 5′-CAAACTTTCTCCTACTT-3′ was identified to overlap with this PPRE motif in six of the seven species (all except for the mouse) for which the enhancer was screened using rVista (Table S1). Conversely, the rat upTRE motif (Rabelo et al., 1995) appears to have arisen from a 14 bp deletion in this species, and is therefore not present in other lineages (Figure 7). Additionally, the white-headed capuchin (*Cebus capuchinis*) and polar bear (*Ursus maritimus*), both of which likely express functional BAT, have deletions within the putative upTRE region. The 5′ portion of the dnTRE motif (5′-AGGGCAGCAAGGTCA-3′) described by Rabelo et al. (1995) is also exclusive to the rat, as the consensus sequence (5′-AG**A**AGGGGTGAGGTCA-3′) has numerous differences and an insertion [bold]; deletions to this region are also found in the Damaraland mole-rat (*Fukomys damarensis*), *Myotis* spp. bats, and the lesser hedgehog tenrec (Figure 7). The NBRE site, which overlaps with the 3′ region of the dnTRE, is not strongly conserved in all species, with nucleotide deletions in artiodactyls, the Damaraland mole-rat, great roundleaf bat (*Hipposideros armiger*), David's myotis, and natal long-fingered bat, and insertions in both the tiger and the giant ground sloth (Figure 7). The most crucial nucleotides of the BRE-2 motif (5′-TTCC-3′; bases 219–222 of the enhancer alignment; Figure 7) described by Kozak et al. (1994) are only found in mice (the species in which it was first described) and the Chinese rufous horseshoe bat (*Rhinolophus sinicus*).

## Discussion

**Figure 9:**
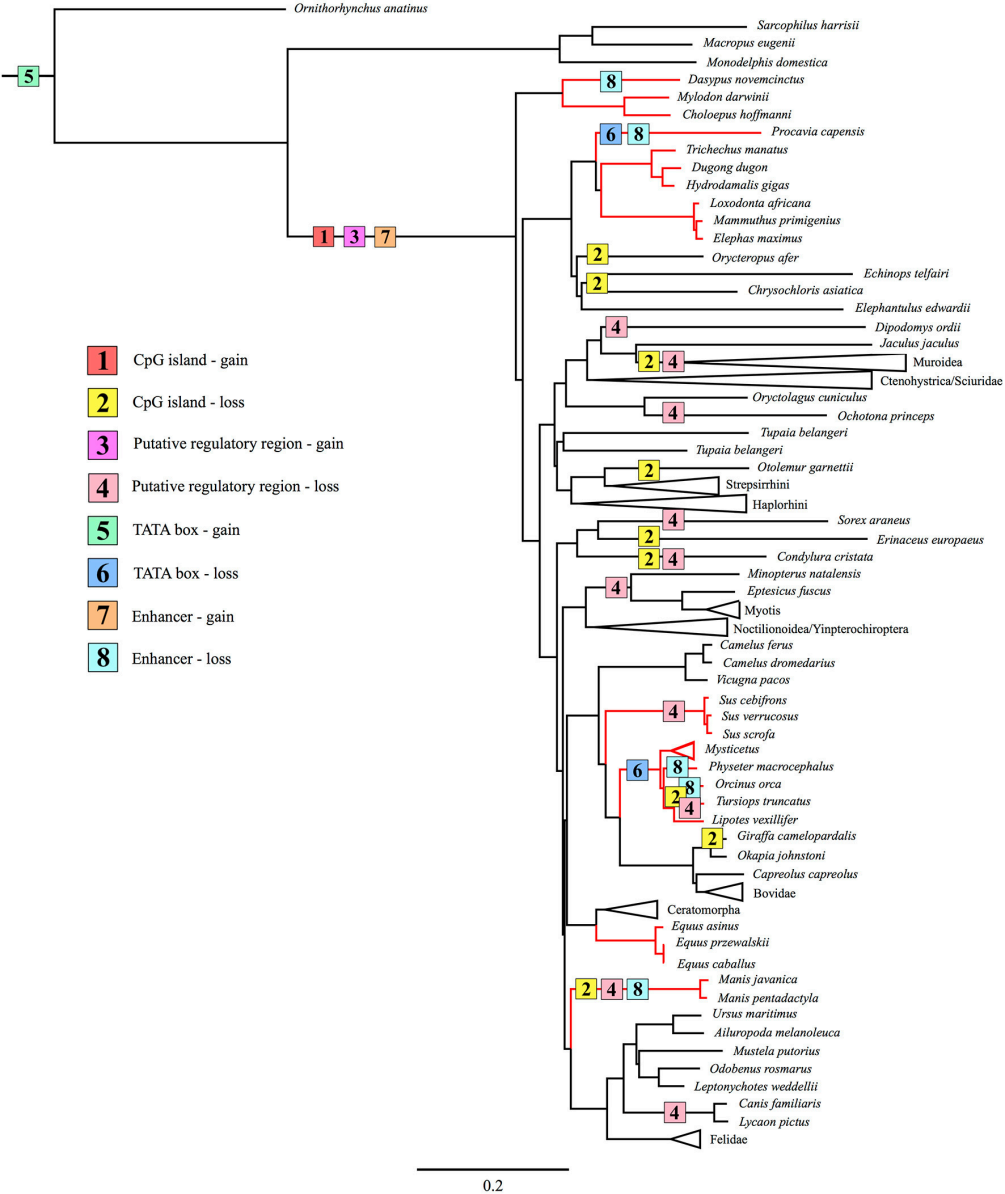
Maximum likelihood species tree based on 41 gene segments (50,911 base pairs) composed of both coding and non-coding regions illustrating the gain and loss of known *UCP1* regulatory elements (CpG island, PRR, TATA box, enhancer) through the evolutionary history of Mammalia. Red branches indicate lineages with a non-functional *UCP1* gene (Gaudry et al., 2017).

No traces of an enhancer, PRR, or CpG island were detected in the upstream region of the platypus or gray short-tailed opossum loci, though both appear to possess a TATA box within the proximal promoter. By contrast, each of these elements were observed in afrotherians, euarchontoglirans, and laurasiatherians, while a portion of the *UCP1* enhancer was also obtained in a single xenarthran, the giant ground sloth, a species that went extinct during the late Pleistocene ~12,000 years ago (Moore, 1978). We can thus deduce that the *UCP1* gene of stem mammals contained a TATA box, while the other transcriptional regulatory elements evolved in a common ancestor of eutherians as proposed by Jastroch et al. (2008). However, despite functioning as a hypothetical methylation site (CpG island) or encompassing putative transcription factor binding sites in some species (PRR), these motifs are not required for BAT transcription, as exemplified by high *UCP1* expression within the BAT of mice and rats (Pedersen et al., 2001; Wu et al., 2012), which lack both of these elements. Indeed, these elements have repeatedly been lost in eutherian mammals (Figure 9). Shore et al. (2012) reached a similar conclusion as roughly half of the eutherian species they examined lacked a PRR and a CpG island. Given the proposed function of the CpG island as a regulator of UCP1 tissue-specific expression (Kiskinis et al., 2007), a lower level of methylation in BAT as opposed to other tissues would be expected, however, Shore et al. (2012) discovered that the *UCP1* CpG island remains virtually un-methylated in BAT, white adipose tissue, and liver despite greatly reduced UCP1 expression levels in the latter two tissues. Therefore, the function of this region remains unclear, however, Shore et al. (2012) did characterize a CpG island in the zebrafish suggesting its presence could be an ancestral condition of the *UCP1* gene that was lost in non-eutherian mammals, but retained (and again lost) in some eutherians (see Figure 9).

Alignment of the proximal promoter CRE-4 site among representative eutherians reveals that the 5′-TGACGCGC-3′ sequence proposed by Kozak et al. (1994) is conserved in the rat, but deviates considerably in the shrew, cow, and human, which are known to express functional BAT (Heaton, 1972; Alexander et al., 1975; Przełecka, 1981). Thus, while the CRE-4 site may play an important role within the murid lineage, it likely does not apply to other eutherians. Similarly, the CCAAT box proposed by Bouillaud et al. (1988) in the rat is highly variable among eutherians (and even among rodents), thus is also unlikely to be a key site for promoter activity. Of the three GCCCCT sites proposed by Yubero et al. (1994), only the two located proximal to exon 1 are conserved, however, to our knowledge transcription factors that bind to these nucleotides have not yet been identified. Overall, the TATA box of the *UCP1* promoter is highly conserved in most eutherians, but does vary in some species. For instance, the shared TACA box variant among the horse, rhino, bats, and panda is interesting given that bats and bears possess discernible BAT (Rowlatt et al., 1971; Thomas et al., 1990). While TATA box variants of the flowering plant *Arabidopsis thaliana*, including the 5′-TACAAAAG-3′ sequence, can still bind the TATA binding protein (TBP) without any structural modifications to the protein, transcription activity levels are substantially (76–85%) lower compared to the 5′-TATAAAAG-3′ sequence (Patikoglou et al., 1999). Considering the high level of TBP conservation among eukaryotes (Peterson et al., 1990), its ability to bind TATA box variants may also apply to mammals. The same T → C transition at the third nucleotide position has been described in the TATA (TACA) box of rabbit uteroglobin with respect to the rat and human, causing a 7-fold reduction in activity when binding to TBP (Klug et al., 1994). However, two other proteins (TATA core factor and TATA palindrome factor) present in uteroglobin-expressing cells bind the TACA box with high efficiency to promote cell specific-expression of the protein (Klug et al., 1994), thus the same possibility may apply to bears, bats, and rhinos. The mutated 5′-TAAATAA-3′ site of the walrus retains a high A/T richness and can thus likely still efficiently bind the TBP (Patikoglou et al., 1999). Notably, the TATA boxes of the hyrax and cetacean *UCP1* pseudogenes are poorly conserved, likely due to mutations accumulating under neutral evolution (Figure 9).

In general, the *UCP1* enhancer appears to be among the most crucial elements of transcriptional regulation as it is one of the few highly conserved regions in the upstream sequence between humans and rodents (Figure 6). Indeed, excluding four species with low sequence coverage (see below), the enhancer was recovered from all eutherians with an intact *UCP1* gene, and therefore is likely essential for *UCP1* expression in BAT. This conclusion is at odds with that of Shore et al. (2012), who incorrectly deduced that this region was deleted in a number of species. While we were unable to retrieve an enhancer in four species (i.e., European hedgehog, zebu, Brazilian guinea pig, and desert woodrat), contigs of these species either do not extend ~5 kb upstream of *UCP1* exon 1 or contain large sequencing gaps.

In concert with our prediction that large body size may be associated with relaxed selection pressures for UCP1 expression, several anomalies among putative transcription factor binding motifs exist that could be indicative of degradation of these elements were observed. For instance, rhinoceroses display a deletion within the BRE-2 site, and multiple mutations occur within the dnTRE and NBRE regions of camels and the alpaca (*Vicugna pacos*). However, deletions also occur within these regions of some small-bodied species (Damaraland mole-rat, lesser hedgehog tenrec, and *Myotis* spp. bats) that also have an intact *UCP1*, while felids display a highly divergent nucleotide sequence within this 3′ region of the enhancer box. Overall, it thus seems unlikely that these transcriptional regulatory element mutations would substantively impact *UCP1* expression in the large-bodied species. Notably, *UCP1* regulatory regions (enhancer, PRR, CpG island, promoter) are also present in all large-bodied species (e.g., rhinoceroses, pinnipeds, camel), except the giraffe where a CpG island was not detected (Table 1). Again, this finding suggests that the UCP1 protein may be present in BAT and/or beige tissue of these lineages, highlighting the need for future investigation of UCP1 expression in these species.

In support of our hypothesis that transcriptional regulators would be deteriorated or lost in eutherians with *UCP1* pseudogenes, at least five independent lineages (sperm whale, hyrax, pangolins, armadillo, and the family delphinidae [killer whale and bottlenose dolphin]) lack an *UCP1* enhancer (Figure 9); notably the TATA box is also lost/mutated in these lineages. By contrast, we identified several lineages (elephantids, sirenians, suids, equids, and some cetaceans) that retain a highly conserved enhancer despite inactivation of their *UCP1* genes >20 MYA (Gaudry et al., 2017). The presence of a conserved enhancer upstream of the pig *UCP1* pseudogene was also noted by Shore et al. (2012), who suggested that an added function might explain its high degree of sequence identity to that of humans. One such added function could be pleiotropy; the regulation multiple genes (He and Zhang, 2006). Indeed, evolutionary constraint increases (i.e., a higher degree of purifying selection) in mammalian enhancers with increasing pleiotropy (Hiller et al., 2012). Considering that pleiotropic enhancers are not uncommon among mammals (Hiller et al., 2012), this hypothesis cannot be entirely discounted. However, the loss of an *UCP1* enhancer in the sperm whale, killer whale, bottlenose dolphin, hyrax, armadillo, and pangolins implies that this enhancer is non-pleiotropic. The apparent conservation of most enhancer elements in the other species for which *UCP1* is pseudogenized (e.g., baleen whales, elephants, sirenians, horses) is presumably in part due to an inherently slow rate of molecular evolution arising from their large body size. Indeed, other pseudogenized genes (e.g., *AMBN, AMEL, ENAM*, and *MMP20*) in baleen whales and the Steller's sea cow (*H. gigas*) show exceptionally low rates of molecular decay (Meredith et al., 2011; Springer et al., 2015). Consequently the high (>80%) enhancer sequence identity shared between *UCP1*-pseudogenized species (horse, minke whale, pig, baiji, bowhead whale, African elephant, and manatee) and humans is not surprising. It thus also remains possible that slow rates of DNA evolution may explain the retention and conservation of these regulatory elements in some large-bodied species with intact *UCP1* CDS. By contrast, the higher sequence divergence in rats and mice, which share only 69 and 74% of *UCP1* enhancer similarity with humans, respectively, can likely be attributed to a relatively fast mutation rate.

Surprisingly, an elevated mutation rate is also evident in the *UCP1* coding sequence of canids as well as the small-bodied lesser hedgehog tenrec, myomorph rodents, vesper bats, and, particularly within members of the order eulipotyphla (Figure 8). While selection pressure analyses indicate that the *UCP1* coding sequences of these species display relatively low dN/dS ratios (<0.22; Gaudry et al., 2017), associated with functional conservation of the protein, the very high substitution rates in these groups equate to a substantively elevated number of non-synonymous amino acid substitutions relative to other eutherian lineages (Figure S1). Notably, these high substitution rates are not found for *UCP2* or *UCP3* sequences of these species (cf. Figure 1), suggesting that this is not solely a size-dependent phenomenon. Consequently these lineages provide intriguing comparative opportunities to study functional UCP1 attributes, as BAT-mediated NST is likely crucial for thermoregulation in these lineages.

A key finding of this study is that several transcription factor binding motifs first described in either mice or rats (BRE-1, BRE-2, upTRE, dnTRE) appear to be restricted to this clade of mammals. Other enhancer motifs (URE1, CRE-2, RARE-2, NBRE) presumed to be key for transcription factor binding in murid rodents (Kozak et al., 1994; Rabelo et al., 1996; Sears et al., 1996; Kumar et al., 2008) are also mutated in other eutherian lineages (Figure 7). Although both single point mutations (Bokar et al., 1988) or combination of mutations (Rabelo et al., 1996) have been shown to alter transcription factor binding to some of these motifs in murid rodents, the effect of the observed differences to these motifs in other eutherians needs to be assessed. Nonetheless, the rVista enhancer screening (Table S1) demonstrates that a number of putative transcription factor binding elements (e.g., CRE-2, PPARG) are not shared between murid rodents and the consensus sequence. This analysis also suggests that components of the transcriptional control of *UCP1* expression may be differentially regulated among eutherian mammals. For example, the CRE-3 element was identified in each species selected for screening except for the star-nosed mole (Table S1). By contrast, the high level of sequence identity of the PPRE and RARE-3 elements across Placentalia (Figure 7) indicates that their function has remained strongly constrained throughout eutherian evolution, and is suggestive that they are universally required for the regulation and specificity of *UCP1* transcription.

## Conclusions

To our knowledge, this study represents the broadest comparative analysis of *UCP1* transcriptional regulatory elements among mammals. Our results demonstrate that the CpG island and PRR are not universally conserved among BAT-expressing eutherians and thus are likely not required for *UCP1* transcription. In contrast, the TATA box and two of the three GCCCCT sites in the promoter are highly conserved and presumably play a transcriptional role, while the CRE-4 and CCAAT sites differ substantially among eutherians and likely are unimportant. While a *UCP1* enhancer was found to be present in every eutherian superorder (Xenarthra [partial], Afrotheria, Laurasiatheria, Euarchontoglires), its absence among non-eutherian mammals supports the hypothesis that it originated with the rise of BAT in a stem placental ancestor. Within this region, however, the specificity and importance of the upTRE, dnTRE, URE1, CRE-2, RARE-2, NBRE, BRE-1, and BRE-2 enhancer elements first described from rats and mice are uncertain as these motifs differ substantially—but generally remain highly conserved—in other BAT-expressing eutherians. Conversely, the RARE-3 and PPRE motifs are among the most highly conserved putative transcription factor binding elements and are likely functional across the eutherian phylogeny. Finally, while some *UCP1*-less species still retain a *UCP1* enhancer, this sequence conservation is presumably due to a slow rate of neutral evolution. Nonetheless, lack of an enhancer in seven *UCP1*-less species strongly suggests this element is non-pleiotropic.

## Author contributions

MG conceived of the project, designed research, prepared DNA libraries, performed hybridization capture experiments, conducted sequencing and genome-mining, performed comparative bioinformatic analyses, prepared the figures, interpreted the results, and drafted the manuscript. KC conceived of the project, designed research, interpreted the results, and revised the manuscript.

### Conflict of interest statement

The authors declare that the research was conducted in the absence of any commercial or financial relationships that could be construed as a potential conflict of interest.

## References

[B1] Alexander G. Bennett J. W. Gemmell R. T. (1975). Brown adipose tissue in the new-born calf (*Bos taurus*). J. Physiol. 244, 223–234. 10.1113/jphysiol.1975.sp010793 1123748 PMC1330754

[B2] Berg F. Gustafson U. Andersson L. (2006). The uncoupling protein 1 gene (UCP1) is disrupted in the pig lineage: a genetic explanation for poor thermoregulation in piglets. PLoS Genet. 2:e129. 10.1371/journal.pgen.0020129 16933999 PMC1550502

[B3] Bianco A. C. Silva J. E. (1987). Optimal response of key enzymes and uncoupling protein to cold inbrown adipose tissue depends on local T_3_ generation. Am. J. Physiol. 253, E255–E263. 3631256 10.1152/ajpendo.1987.253.3.E255

[B4] Bokar J. A. Roesler W. J. Vandenbark G. R. Kaetzel D. M. Hanson R. W. Nilson J. H. (1988). Characterization of the cAMP responsive elements from the genes for the alpha-subunit of glycoprotein hormones and phosphoenolpyruvate carboxykinase (GTP). Conserved features of nuclear protein binding between tissues and species. J. Biol. Chem. 263, 19740–19747. 2461942

[B5] Bouillaud F. Raimbault S. Ricquier D. (1988). The gene for rat uncoupling protein: complete sequence, structure of primary transcript and evolutionary relationship between exons. Biochem. Biophys. Res. Commun. 157, 783–792. 10.1016/S0006-291X(88)80318-8 3202878

[B6] Brand M. D. Esteves T. C. (2005). Physiological functions of the mitochondrial uncoupling proteins UCP2 and UCP3. Cell Metab. 2, 85–93. 10.1016/j.cmet.2005.06.002 16098826

[B7] Brent G. A. Moore D. D. Larsen R. P. (1991). Thyroid hormone regulation of gene expression. Annu. Rev. Physiol. 53, 17–35. 10.1146/annurev.ph.53.030191.000313 2042958

[B8] Cannon B. Nedergaard J. (2004). Brown adipose tissue: function and physiological significance. Physiol. Rev. 84, 277–359. 10.1152/physrev.00015.2003 14715917

[B9] Echtay K. (2007). Mitochondrial uncoupling proteins-What is their physiological role? Free Radic. Biol. Med. 43, 1351–1371. 10.1016/j.freeradbiomed.2007.08.011 17936181

[B10] Edgar R. C. (2004). MUSCLE: multiple sequence alignment with high accuracy and high throughput. Nucleic Acids Res. 19, 1792–1797. 10.1093/nar/gkh340 PMC390337 15034147

[B11] Gardiner-Garden M. Frommer M. (1987). CpG islands in vertebrate genomes. J. Mol. Biol. 196, 261–282. 10.1016/0022-2836(87)90689-9 3656447

[B12] Gaudry M. J. Jastroch M. Treberg J. R. Hofreiter M. Paijmans J. L. A. Starret J.. (2017). Inactivation of thermogenic UCP1 as a historical contingency in multiple placental mammal clades. Sci. Adv. 3:e1602878. 10.1126/sciadv.1602878 28706989 PMC5507634

[B13] Harms M. Seale P. (2013). Brown and beige fat: development, function and therapeutic potential. Nat. Med. 19, 1252–1263. 10.1038/nm.3361 24100998

[B14] He X. Zhang J. (2006). Toward a molecular understanding of pleiotropy. Genetics 173, 1885–1891. 10.1534/genetics.106.060269 16702416 PMC1569710

[B15] Heaton J. M. (1972). The distribution of brown adipose tissue in the human. J. Anat. 112, 35–39. 5086212 PMC1271341

[B16] Heldmaier G. (1971). Nonshivering thermogenesis and body size in mammals. J. Comp. Physiol. 73, 222–248.

[B17] Herpin P. Damon M. Le Dividich J. (2002). Development of thermoregulation and neonatal survival in pigs. Livest. Prod. Sci. 78, 25–45. 10.1016/S0301-6226(02)00183-5

[B18] Hiller M. Schaar B. T. Bejerano G. (2012). Hundreds of conserved non-coding genomic regions are independently lost in mammals. Nucleic Acids Res. 40, 11463–11476. 10.1093/nar/gks905 23042682 PMC3526296

[B19] Hughes D. A. Jastroch M. Stoneking M. Klingenspor M. (2009). Molecular evolution of UCP1 and the evolutionary history of mammalian non-shivering thermogenesis. BMC Evol. Biol. 9:4. 10.1186/1471-2148-9-4 19128480 PMC2627829

[B20] Jastroch M. Buckingham J. A. Helwig M. Klingenspor M. Brand M. D. (2007). Functional characterization of UCP1 in the common carp: uncoupling activity in liver mitochondria and cold-induced expression in the brain. J. Comp. Physiol. B Biochem. Syst. Environ. Physiol. 177, 743–752. 10.1007/s00360-007-0171-6 17576568

[B21] Jastroch M. Withers K. W. Taudien S. Frappell P. B. Helwig M. Fromme T.. (2008). Marsupial uncoupling protein 1 sheds light on the evolution of mammalian nonshivering thermogenesis. Physiol. Genomics 32, 161–169. 10.1152/physiolgenomics.00183.2007 17971503

[B22] Kiskinis E. Hallberg M. Christian M. Olofsson M. Dilworth S. M. White R.. (2007). RIP140 directs histone and DNA methylation to silence Ucp1 expression in white adipocytes. EMBO J. 26, 4831–4840. 10.1038/sj.emboj.7601908 17972916 PMC2099470

[B23] Klingenspor M. Fromme T. (2012). Brown adipose tissue, in Adipose Tissue Biology, ed Symonds M. E. (New York, NY: Springer), 39–79.

[B24] Klingenspor M. Fromme T. Hughes D. A. Manzke L. Polymeropoulos E. Riemann T.. (2008). An ancient look at UCP1. Biochim. Biophys. Acta 1777, 637–641. 10.1016/j.bbabio.2008.03.006 18396149

[B25] Klug J. Knapp S. Castro I. Beato M. (1994). Two distinct factors bind to the rabbit uteroglobin TATA-box region and are required for efficient transcription. Mol. Cell. Biol. 14, 6208–6218. 10.1128/MCB.14.9.6208 8065353 PMC359148

[B26] Kozak U. C. Kopecky J. Teisinger J. Enerback S. Boyer B. Kozak L. P. (1994). An upstream enhancer regulating brown-fat-specific expression of the mitochondrial uncoupling protein gene. Mol. Cell. Biol. 14, 59–67. 8264627 10.1128/mcb.14.1.59-67.1994 PMC358356

[B27] Kumar N. Liu D. Wang H. Robidoux J. Collins S. (2008). Orphan nuclear receptor NOR-1 enhances 3′,5′-cyclic adenosine 5′-monophosphate-dependent uncoupling protein-1 gene transcription. Mol. Endocrinol. 22, 1057–1064. 10.1210/me.2007-0464 18238829 PMC2366189

[B28] Lin J. Cao C. Tao C. Ye R. Dong M. Zheng Q.. (2017). Cold adaptation in pigs depends on UCP3 in beige adipocytes. J. Mol. Cell. Biol. [Epub ahead of print]. 10.1093/jmcb/mjx018 28486585

[B29] Loots G. Ovcharenko I. (2004). rVista 2.0: evolutionary analysis of transcription factor binding sites. Nucleic Acids Res. 32, W217–W221. 10.1093/nar/gkh383 15215384 PMC441521

[B30] Mailloux R. J. Harper M. E. (2011). Uncoupling proteins and the control of mitochondrial reactive oxygen species production. Free Radic. Biol. Med. 51, 1106–1115. 10.1016/j.freeradbiomed.2011.06.022 21762777

[B31] Mantovani R. (1999). The molecular biology of the CCAAT-binding factor NF-Y. Gene 239, 15–27. 10.1016/S0378-1119(99)00368-6 10571030

[B32] McGaugh S. Schwartz T. S. (2017). Here and there, but not everywhere: repeated loss of uncoupling protein 1 in amniotes. Biol. Lett. 13:20160749 10.1098/rsbl.2016.0749 28052937 PMC5310580

[B33] McNab B. K. (1983). Energetics, body size, and the limits to endothermy. J. Zool. 199, 1–29. 10.1111/j.1469-7998.1983.tb06114.x

[B34] Meredith R. W. Gatesy J. Cheng J. Springer M. S. (2011). Pseudogenization of the tooth gene enamelysin (*MMP20*) in the common ancestor of baleen whales. Proc. Biol. Soc. 278, 993–1002. 10.1098/rspb.2010.1280 20861053 PMC3049022

[B35] Meyer M. Kircher M. (2010). Illumina sequencing library preparation for highly multiplexed target capture and sequencing. Cold Spring Harb. Protoc. 6:Pbdprot5448. 10.1101/pdb.prot5448 20516186

[B36] Moore D. I. (1978). Post-glacial vegetation in the South Patagonian territory of the giant ground sloth, Mylodon. Bot. J. Linean. Soc. 77, 177–202. 10.1111/j.1095-8339.1978.tb01398.x

[B37] Mzilikazi N. Jastroch M. Meyer C. W. Klingenspor M. (2007). The molecular and biochemical basis of nonshivering thermogenesis in an African endemic mammal, *Elephantulus myurus*. Am. J. Physiol. Regul. Integr. Comp. Physiol. 293, R2120–R2127. 10.1152/ajpregu.00427.2007 17686883

[B38] Nakajima N. Horikoshi M. Roeder R. G. (1988). Factors involved in specific transcription by mammalian RNA polymerase II: purification, genetic specificity, and TATA box-promoter interactions of TFIID. Mol. Cell. Biol. 8, 4028–4040. 10.1128/MCB.8.10.4028 3185540 PMC365471

[B39] Oelkrug R. Goetze N. Exner C. Lee Y. Ganjam G. K. Kutschke M.. (2013). Brown fat in a protoendothermic mammal fuels eutherian evolution. Nat. Commun. 4:2140. 10.1038/ncomms3140 23860571 PMC3717499

[B40] Oelkrug R. Polymeropoulos E. T. Jastroch M. (2015). Brown adipose tissue: physiological function and evolutionary significance. J. Comp. Physiol. B Biochem. Syst. Environ. Physiol. 185, 587–606. 10.1007/s00360-015-0907-7 25966796

[B41] Patikoglou G. A. Kim J. L. Sun L. Yang S. H. Kodadek T. Burley S. K. (1999). TATA element recognition by the TATA box-binding protein has been conserved throughout evolution. Genes Dev. 13, 3217–3230. 10.1101/gad.13.24.3217 10617571 PMC317201

[B42] Pearson L. E. Liwanag H. E. Hammill M. O. Burns J. M. (2014). To each its own: thermoregulatory strategy varies among neonatal polar phocids. Comp. Biochem. Physiol. A Mol. Integr. Physiol. 178, 59–67. 10.1016/j.cbpa.2014.08.006 25151642

[B43] Pedersen S. B. Bruun J. M. Kristensen K. Richelsen B. (2001). Regulation of UCP1, UCP2, and UCP3 mRNA expression in brown adipose tissue, white adipose tissue, and skeletal muscle in rats by estrogen. Biochem. Biophys. Res. Commun. 288, 191–197. 10.1006/bbrc.2001.5763 11594772

[B44] Peterson M. G. Tanese N. Pugh B. F. Tjian R. (1990). Functional domains and upstream activation properties of cloned human TATA binding protein. Science 248, 1625–1630. 10.1126/science.249.4971.844-b 2363050

[B45] Polymeropoulos E. T. Jastroch M. Frappell P. B. (2012). Absence of adaptive nonshivering thermogenesis in a marsupial, the fat-tailed dunnart (*Sminthopsis crassicaudata*). J. Comp. Physiol. B Biochem. Syst. Environ. Physiol. 182, 393–401. 10.1007/s00360-011-0623-x 22002052

[B46] Przełecka A. (1981). Seasonal changes in ultrastructure of brown adipose tissue in the common shrew (*Sorex araneus L*). Cell Tissue Res. 214, 623–632. 10.1007/BF00233501 7214470

[B47] Rabelo R. Reyes C. Schifman A. Silva J. E. (1996). A complex retinoic acid response element in the uncoupling protein gene defines a novel role for retinoids in thermogenesis. Endocrinology 137, 3488–3496. 8754778 10.1210/endo.137.8.8754778

[B48] Rabelo R. Schifman A. Rubio A. Sheng X. Silva J. E. (1995). Delineation of thyroid hormone-responsive sequences within a critical enhancer in the rat uncoupling protein gene. Endocrinology 136, 1003–1013. 10.1210/endo.136.3.7867554 7867554

[B49] Rowlatt U. Mrosovsky N. English A. (1971). A comparative survey of brown fat in the neck and axilla of mammals at birth. Biol. Neonate 17, 53–83. 10.1159/000240303 5549489

[B50] Saito S. Saito C. T. Shingai R. (2008). Adaptive evolution of the uncoupling protein 1 gene contributed to the acquisition of novel nonshivering thermogenesis in ancestral eutherian mammals. Gene 408, 37–44. 10.1016/j.gene.2007.10.018 18023297

[B51] Sears I. B. MacGinnitie M. A. Kovacs L. G. Graves R. A. (1996). Differentiation-dependent expression of the brown adipocyte uncoupling protein gene: regulation by peroxisome proliferator-activated receptor gamma. Mol. Cell. Biol. 16, 3410–3419. 10.1128/MCB.16.7.3410 8668156 PMC231335

[B52] Shore A. Emes R. D. Wessely F. Kemp P. Cillo C. D'Armiento M.. (2012). A comparative approach to understanding tissue-specific expression of uncoupling protein 1 expression in adipose tissue. Front. Genet. 3:304. 10.3389/fgene.2012.00304 23293654 PMC3535714

[B53] Shore A. Karamitri A. Kemp P. Speakman J. R. Lomax M. A. (2010). Role of UCP1 enhancer methylation and chromatin remodeling in the control of UCP1 expression in murine adipose tissue. Diabetologia 51, 1164–1173. 10.1007/s00125-010-1701-4 PMC2860566 20238096

[B54] Springer M. S. Signore A. V. Paijmans J. L. Vélez-Juarbe J. Domning D. P. Bauer C. E.. (2015). Interordinal gene capture, the phylogenetic position of Steller's sea cow based on molecular and morphological data, and the macroevolutionary history of Sirenia. Mol. Phylogenet. Evol. 91, 178–193. 10.1016/j.ympev.2015.05.022 26050523

[B55] Stamatakis A. (2006). RAxML-VI-HPC: maximum likelihood-based phylogenetic analyses with thousands of taxa and mixed models. Bioinformatics 22, 2688–2690. 10.1093/bioinformatics/btl446 16928733

[B56] Sullivan M. J. Petty N. K. Beatson S. A. (2011). Easyfig: a genome comparison visualizer. Bioinformatics 27, 1009–1010. 10.1093/bioinformatics/btr039 21278367 PMC3065679

[B57] Thomas D. W. Dorais M. Bergeron J. M. (1990). Winter energy budgets and cost of arousals for hibernating little brown bats, *Myotis lucifugus*. J. Mamm. 71, 475–479. 10.2307/1381967

[B58] Umesono K. Murakami K. K. Thompson C. C. Evans R. M. (1991). Direct repeats as selective response elements for the thyroid hormone, retinoic acid, and vitamin D3 receptors. Cell 65, 1255–1266. 10.1016/0092-8674(91)90020-Y 1648450 PMC6159884

[B59] Villarroya F. Peyrou M. Giralt M. (2017). Transcriptional regulation of the uncoupling protein-1 gene. Biochimie 134, 86–92. 10.1016/j.biochi.2016.09.017 27693079

[B60] Wu J. Boström P. Sparks L. M. Ye L. Choi J. H. Giang A. H.. (2012). Beige adipocytes are a distinct type of thermogenic fat cell in mouse and human. Cell 150, 366–376. 10.1016/j.cell.2012.05.016 22796012 PMC3402601

[B61] Xu L. Thali M. Schaffner W. (1991). Upstream box/TATA box order is the major determinant of the direction of transcription. Nucleic Acids Res. 19, 6699–6704. 10.1093/nar/19.24.6699 1762900 PMC329297

[B62] Yubero P. Vinas O. Iglesias R. Mampel T. Villarroya F. Giralt M. (1994). Identification of tissue-specific protein binding domains in the 5'-proximal regulatory region of the rat mitochondrial brown fat uncoupling protein gene. Biochem. Biophys. Res. Commun. 204, 867–873. 10.1006/bbrc.1994.2540 7980554

