# Supplementary figures and images for: Evolution of UCP1 Transcriptional Regulatory Elements Across the Mammalian Phylogeny
Source: Front Physiol. 2017 Sep 20;8:670. doi: 10.3389/fphys.2017.00670 (PMC5611445; doi:10.3389/fphys.2017.00670)

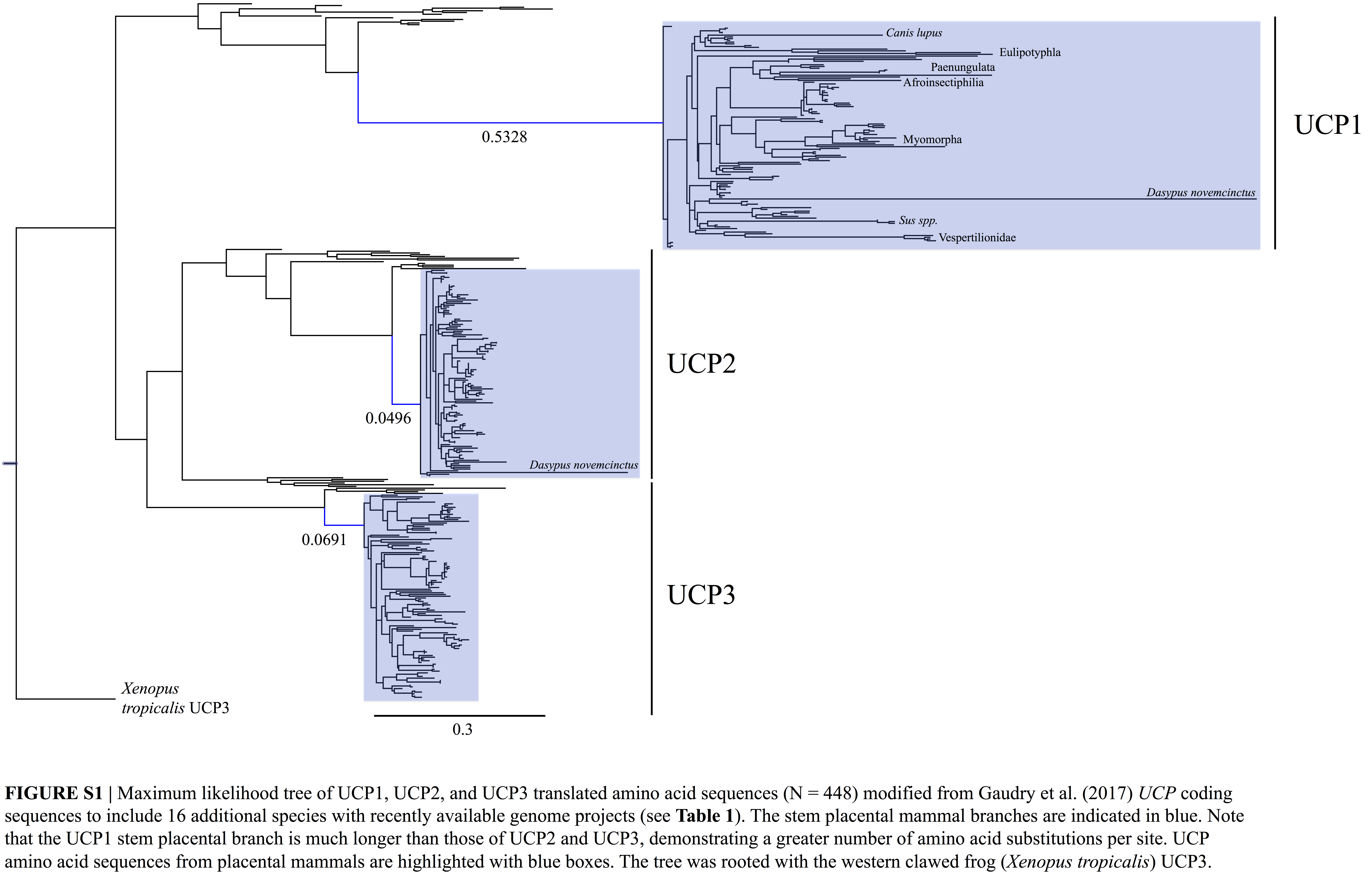

Supplement: Supplementary file 2 [file Image1.tiff]
