# Supplementary material for: Evolution of UCP1 Transcriptional Regulatory Elements Across the Mammalian Phylogeny
Source: Front Physiol. 2017 Sep 20;8:670. doi: 10.3389/fphys.2017.00670 (PMC5611445; doi:10.3389/fphys.2017.00670)
Supplement: Supplementary file 4 [file DataSheet2.DOCX]

DNA, A2AB = 1-1203

DNA, ADRB2 = 1204-2013

DNA, APP = 2014-2700

DNA, ATP7A = 2701-3384

DNA, Adora3 = 3385-3729

DNA, ApoB = 3730-6240

DNA, BCHE = 6241-7230

DNA, BDNF = 7231-7794

DNA, BMI1 = 7795-8086

DNA, BRCA1 = 8087-10933

DNA, BRCA2 = 10934-15640

DNA, CHRNA1 = 15641-16048

DNA, CMYC = 16049-16642

DNA, CNR1 = 16643-17635

DNA, CREM = 17636-18078

DNA, DMP1 = 18079-19458

DNA, ENAM = 19459-22380

DNA, EDG1 = 22381-23337

DNA, FBN1 = 23338-23951

DNA, GHR = 23952-24710

DNA, IRBP = 24711-25990

DNA, MC1R = 25991-26941

DNA, Mt_rRNA = 26942-28829

DNA, Mt_protein = 28830-38474

DNA, PLCB4 = 38475-38836

DNA, PNOC = 38837-39178

DNA, Rag1 = 39179-42217

DNA, Rag2 = 42218-43582

DNA, SWS1 = 43583-44629

DNA, TTN = 44630-49084

DNA, TYR1 = 49085-49651

DNA, VWF = 49652-50911
